# Vascular surgery in liver resection

**DOI:** 10.1007/s00423-021-02310-w

**Published:** 2021-09-14

**Authors:** Olga Radulova-Mauersberger, Jürgen Weitz, Carina Riediger

**Affiliations:** 1 Department of Visceral, Thoracic and Vascular Surgery, University Hospital Carl Gustav Carus, Technische Universität Dresden, Fetscherstraße 74, 01307 Dresden, Germany; 2 National Center for Tumor Diseases (NCT/UCC), Dresden, Germany; 3 German Cancer Research Center (DKFZ), Heidelberg, Germany; 4 Faculty of Medicine and University Hospital Carl Gustav Carus, Technische Universität Dresden, Dresden, Germany; 5 Helmholtz-Zentrum Dresden - Rossendorf (HZDR), Dresden, Germany

**Keywords:** Hepatectomy, Vena cava resection, Portal interposition, Caval shift, Liver tumors, Liver arteries

## Abstract

Vascular surgery in liver resection is a standard part of liver transplantation, but is also used in oncological liver surgery. Malignant liver tumors with vascular involvement have a poor prognosis without resection. Surgery is currently the only treatment to provide long-term survival in advanced hepatic malignancy. Even though extended liver resections are increasingly performed, vascular involvement with need of vascular reconstruction is still considered a contraindication for surgery in many institutions. However, vascular resection and reconstruction in liver surgery—despite being complex procedures—are safely performed in specialized centers. The improvements of the postoperative results with reduced postoperative morbidity and mortality are a result of rising surgical and anesthesiological experience and advancements in multimodal treatment concepts with preconditioning measures regarding liver function and systemic treatment options. This review focuses on vascular surgery in oncological liver resections. Even though many surgical techniques were developed and are also used during liver transplantation, this special procedure is not particularly covered within this review article. We provide a summary of vascular reconstruction techniques in oncological liver surgery according to the literature and present also our own experience. We aim to outline the current advances and standards in extended surgical procedures for liver tumors with vascular involvement established in specialized centers, since curative resection improves long-term survival and shifts palliative concepts to curative therapy.

## Introduction

Surgery is the only curative treatment for primary and secondary liver malignancies providing the best chances of cure and the lowest local recurrence rates compared to other (local) treatment modalities. Many limitations for liver surgery have been overcome during the last decades due to multimodal treatment concepts with staged liver resections, interventional and systemic therapies, and refinement of surgical techniques. Approximately 50% of the patients with colorectal carcinoma develop liver metastases. Colorectal liver metastases (CRLM) are responsible for 60 to 70% of mortality in this cohort. In addition, primary liver cancer as hepatocellular carcinoma (HCC) and intrahepatic (iCCC) and perihilar cholangiocarcinoma (phCCC) show rising incidences in recent years [1].

Vascular invasion is often considered a contraindication for hepatic surgery in many institutions leading to palliative concepts for the affected patients. One reason is the concern of a dismal prognosis, the other reason is the concern regarding technical resectability if several vessels are infiltrated by the tumor. The primary goal in oncological surgery is the achievement of complete tumor clearance while maintaining patient safety.

Vascular resection and reconstruction are standard procedures in liver transplantation for more than 50 years and those techniques improved constantly since the first liver transplantation by Thomas Starzl in 1963. However, vascular reconstruction in oncological liver resections is often much more demanding because the vessels to be reconstructed are smaller and mostly located intrahepatically with difficult surgical access [2]. Moreover, a combination of loss of liver parenchyma with associated perioperative risks such as postoperative liver failure due to a small liver remnant and vascular reconstruction lead many surgeons to abandon resection in such advanced cases.

Nevertheless, vascular resections and reconstructions are feasible for hepatic arteries, portal veins, hepatic veins, and vena cava. Complex vascular reconstructions can be performed under total vascular exclusion of the liver (TVE) with hypothermic perfusion as in situ, ante situm, or ex situ resections [3, 4].

Although several vascular reconstruction techniques in liver surgery have been described decades ago, improved outcomes in specialized centers with reduced perioperative morbidity and mortality made vascular resection these days more frequent [5, 6].

The current article gives an overview of vascular resection and reconstruction techniques in liver surgery based on the literature and our own experience. We describe vascular resection and reconstruction in extended liver surgery focusing mainly on operative techniques and further on patient selection, perioperative management, morbidity, and mortality.

## Methods

A systematic search of the literature on PubMed, Web of Science, and Cochrane Library, focusing on vascular resection techniques in liver surgery, was performed to identify relevant studies and reviews. MESH (Medical Subject Headings) terms “liver surgery,” “vascular resection,” “liver resection,” “vascular reconstruction,” and the combination of these terms were used for the systematic search between 2000 and 2020. We found 397 relevant publications. Abstracts were identified and reviewed and articles concerning vascular resection with some form of hepatectomy were screened for relevance. Studies in English and German were included [7]**.** After removing duplicates and assessing for eligibility, 87 publications remained for review. In addition, more specialized search terms such as “reconstruction of the hepatic artery,” “reconstruction of the portal vein,” “reconstruction of hepatic veins,” “reconstruction of the inferior vena cava,” “liver surgery,” “hepatectomy,” and combination of these terms were used as well as “arterial and venous reconstruction in visceral and abdominal surgery” without time limitation.

This review aims to show up a summary of surgical standard techniques of vascular surgery in liver resections. An overview of currently available literature on vascular resection and reconstruction techniques in liver surgery is provided. In addition, relevant and highly cited (historical) articles from the twentieth century about vascular reconstructions and liver resections are included as well.

## Patient selection and resection planning

### Preoperative evaluation of resectability

For evaluation of resectability and resection planning, the extent of the tumor and the involvement of vascular structures as well as exact knowledge of the vascular anatomy and possible anatomical variations is essential. The standard diagnostic tool is a contrast-medium enhanced multidetector high-resolution computer tomography (CT). Appropriate protocols with slices of 0.5–1 mm with arterial and venous enhancement allow the interpretation of intrahepatic vascular anatomy and the volume of the hepatic segments [8]. In addition, contrast-medium enhanced magnetic resonance imaging (MRI) with magnetic resonance cholangio-pancreatography (MRCP) is recommended in case of malignancies involving the biliary tract allowing imaging of the tumor involvement and the display of the biliary anatomy. For extended resections, segmentation of the liver and volumetry of the future liver remnant (FLR) is recommended. Three-dimensional simulation technology, developed in Germany in the early 2000s, may also allow a better visualization of the blood vessels and calculation of the volume [9].

**Fig. 1:**
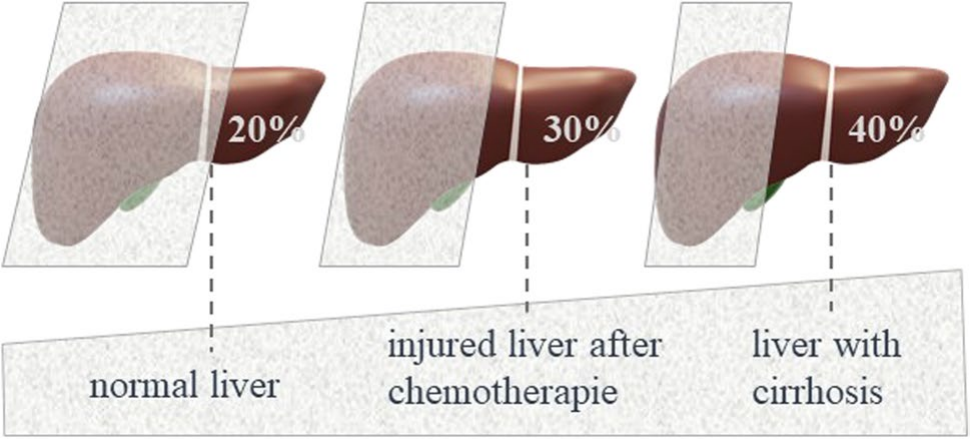
Recommended volume of the future liver remnant (FLR) before liver resection depending on quality/alteration of the liver parenchyma to avoid postoperative liver failure

Some basic factors need to be considered for resection planning:

1. *Inflow*: Inflow structures of the liver are the hepatic artery (HA)and the portal vein (PV). For resection planning, at least one corresponding arterial branch and one portal-venous branch for the remaining liver tissue needs to be preserved or reconstructed [10].
2. *Outflow*: Vascular outflow structures of the liver are hepatic veins (HV). At least one hepatic vein of the remaining liver segment(s) needs to be preserved or reconstructed [11].
  Non-vascular outflow structures are bile ducts; similar to vascular reconstruction, the biliary drainage of the remaining liver needs to be preserved or reconstructed.
3. *Volume of the future liver remnant (FLR)*: A minimum of 20–30% of functional, healthy liver tissue is necessary to maintain liver function and to avoid acute or chronic postoperative liver failure or small for size syndrome [12]. In case of altered liver tissue, preservation of at least 40% or more of the initial liver volume is strongly recommended [13] (Fig. 1). Some authors recommend a FLR of 40% in case of simultaneous vascular reconstruction [14].
  The volume of the FLR can be obtained by CT-based segmentation of the liver and volumetry of the FLR. Volumetry is mostly needed before performing extended liver resections to avoid postoperative liver failure.
4. *Liver parenchyma/quality of liver parenchyma*: The quality of liver parenchyma determines strongly the extent of resection. Many patients undergoing liver resections have underlying liver diseases such as steatosis, (non)-alcoholic steatohepatitis ((N)ASH), liver fibrosis, or liver cirrhosis. In addition, tumor-associated cholestasis or chemotherapy-associated damages as chemotherapy-associated steatohepatitis (CASH) or sinusoidal obstruction syndrome (SOS) are frequent. Altered liver parenchyma has not only reduced reserve capacity to maintain sufficient liver function after resection, but also significantly lowered regenerative capacity with insufficient hypertrophy and the risk of chronic liver failure. This has to be taken into account when estimating the future liver remnant. Noteworthy, 20–30% of fully functioning FLR is necessary to maintain sufficient liver function. Imaging, laboratory tests, and detailed anamnesis help to diagnose liver parenchyma damage.
5. *Liver function*: For the assessment of liver function laboratory tests, clinical scoring systems and specific tests for the measurement of hepatic metabolism are available. Important laboratory tests to estimate liver function are Quick’s value or the international normalized ratio (INR), bilirubin, number of platelets, and albumin. Those laboratory tests are also reflected in the albumin-bilirubin (ALBI) grade or the ALPlat (albumin × platelets) criterion. Similarly, those tests are integrated into the Child–Pugh and MELD (model of end-stage liver disease) score to assess liver function in patients with liver cirrhosis.
  Some authors prefer the use of more specific metabolic tests to estimate a patient’s liver function. Currently available tests to obtain liver function are the indocyanine green (ICG) clearance test, the monoethylglycinexylidide (MEGX) test, or the liver maximum capacity (LiMAX) test [15].
6. *Patient comorbidities*: Besides the oncological frame that should always aim to achieve complete tumor clearance, patient’s age and comorbidities need to be considered when extended liver resections with vascular reconstruction are planned. Especially cardiopulmonary and renal diseases are important for the assessment of the operative risk.

### Preoperative management

#### Preoperative procedures inducing liver hypertrophy of the FLR

In the case of critically small FLR, induction of hypertrophy of the FLR is indicated. Portal vein ligation (PVL) or portal vein embolization (PVE) of the part of the liver to be removed is performed. Noteworthy, PVE or PVL should only be performed with the absence of tumor nodules or complete tumor clearance of the FLR. Consequently, two-staged hepatectomy is necessary in some cases. First reports on this technique were published by Makuuchi et al. [16] and Nagino et al. [17] and were promptly adopted worldwide to reduce the risk of postoperative liver dysfunction. PVE and PVL were compared in a trial showing a slightly higher efficacy of PVE compared to PVL but both methods are feasible and safe [18–20]. In case of insufficient hypertrophy after PVE, preoperative management can be extended with embolization of the corresponding hepatic vein. This method referred to as liver venous deprivation (LVD) can attain a volume increase of the FLR comparable to ALPPS (associating liver partition and portal vein ligation for staged hepatectomy; see below) [21].

ALPPS is a procedure for two-staged liver resection first published as a multicenter experience by the liver surgery group led by Professor Hans Schlitt in Regensburg, Germany [22]. This technique adds splitting of liver parenchyma to the portal vein ligation and induces liver hypertrophy in a short period of 7–14 days of about 60–90% [22, 23]. In our own experience, ALPPS is often used in the case of bilobar tumors with clearance of one side and completion hepatectomy 7–10 days later.

Another possibility to increase the FLR is the use of selective internal radiation treatment (SIRT) for simultaneous induction of hypertrophy due to portal/periportal fibrosis and local tumor control [24]. This approach is only used in few centers. Our experience with this method is limited as possible side effects of radiation on the healthy liver side are not yet clearly evaluated and hypertrophy of the FLR is less compared to the other techniques described above [25].

#### Biliary drainage

Cholestasis has a negative impact on liver regeneration and hypertrophy. In order to reduce the risk of postoperative liver failure after extended hepatectomy, a preoperative biliary drainage (percutaneous and endoscopic drainage) of the FLR for patients with jaundice is a suggested strategy [26–29]. An unilateral drainage of the remnant lobe is recommended and bilateral biliary drainage is considered only for cases with prolonged cholangitis [30]. Preoperative antibiotic treatment of cholangitis is strongly recommended. Some authors describe a significantly reduced risk for postoperative morbidity if preoperative bilirubin can be lowered to 2 mg/dl [31]. In our own experience, it is not necessary to wait for complete normalization of the bilirubin levels as long as cholestasis is regressive and cholangitis has completely resolved.

#### Diagnostic of potential autologous vascular grafts for reconstruction

For proper preoperative planning, evaluation of possible autologous vascular grafts for vascular reconstruction is essential. Ultrasound of the venous system of the lower extremities, especially the greater saphenous vein on both sides including measuring of their diameters, should be performed as well as an ultrasound of external and internal jugular veins.

## Principles of vascular reconstruction in liver surgery

In general, all hepatic vessels can be resected and reconstructed simultaneously in liver surgery. However, morbidity and mortality are significantly rising with the number of reconstructed hepatic vessels (and bile ducts).

Noteworthy, the lowest risk after vascular resection in liver surgery is in case that vascular reconstruction is not necessary due to anatomical variation. Exact knowledge of anatomy may help to avoid reconstruction of vessels in some instances, even if tumors are infiltrating main hilar structures or hepatic veins. A frequent example is the presence of a dominant inferior hepatic vein, draining the posterior sector of the liver (liver segments VI and VII). In those patients, extended left hepatectomy including resection of the left, middle, and right hepatic veins without reconstruction can be performed if the left, middle, and right hepatic veins are infiltrated by the tumor. Another example is the case of an aberrant left hepatic artery originating from the left gastric artery. Arterial reconstruction is often not necessary for extended right hepatectomy even if the tumor is infiltrating central hilar structures.

**Fig. 2:**
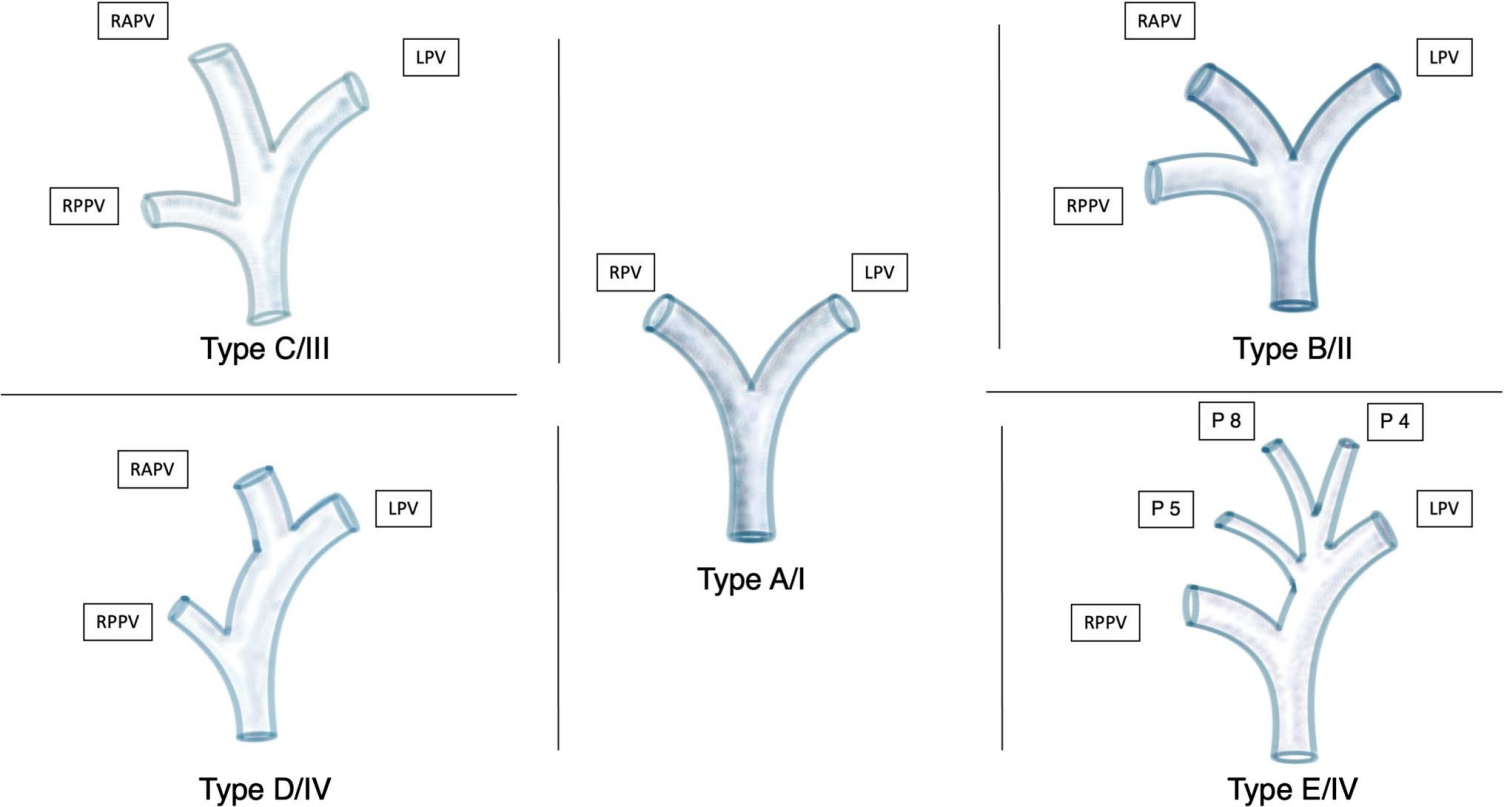
Classification of the portal vein (PV) according to the classifications proposed by Nakamura et al. [32] (Type A to E) and Cheng et al. [33] (Type I to IV). Type A/I Normal anatomy (common variant): bifurcation of the main portal vein (PV) into the left portal vein (LPV) and right portal vein (RPV) (80%) [34]. Type B/II Anatomic variation of a trifurcation of the main PV into the LPV, the right anterior portal vein (RAPV), and the right posterior portal vein (RPPV). A common RPV is missing (7–16%) [8]. Type C/III RPPV arises separately from the main portal vein followed by extraparenchymal bifurcation into the RAPV and LPV intraparenchymal (5%). Type D/IV: RPPV arises separately from the main portal vein followed by intraparenchymal branching of the RAPV. Type E/IV: Separate portal vein branches for liver segments 4, 5, and 8

Important anatomical variations of the portal vein, the hepatic artery, and the hepatic veins are displayed in the related sections hereinafter (Figs. 2, 5, and 6).

Techniques of vascular reconstruction in liver surgery are following general principles in vascular surgery. If possible, reconstruction of vessels should be performed by primary anastomosis. In some cases, primary anastomosis is not possible and graft interposition or use of patches is needed.

In liver surgery, autologous material as patients’ own vessels or peritoneum is preferred. This is especially related to the fact that biliary leakage (with often contaminated bile) is a common complication after liver resection—especially if simultaneous hepaticojejunostomy is performed. The risk of graft infection of autologous materials is significantly lower compared to synthetic materials.

Autologous veins used for vascular reconstruction in liver surgery are the greater saphenous vein, the internal jugular vein, the gonadal vein, and the left renal vein as well as the iliac vein or the splenic vein (Table 1). Peritoneal patches can be used for venous reconstruction as well.

**Table 1:** Grafts and patches for different vascular reconstructions in liver surgery

Vascular reconstruction of	Diameter	Autologous material		Homografts (deceased donor)	Xenogenous material	Synthetic material	
		**Patches**	**Grafts**	**Patches/grafts**	**Patches/grafts**	**Patches**	**Grafts**
**Hepatic artery**	3–6 mm	- Greater saphenous vein - Gonadal vein - Peritoneum	- Greater saphenous vein [35, 36] - Gonadal vein [37] - Left gastric artery [38, 39] - Splenic artery [35, 38, 39] - Inferior mesenteric artery [40, 41] - Radial artery [39] *Transposition of* [42] - Gastroduodenal artery - Left gastric artery - Splenic artery	Cryopreserved Iliac artery [43, 44]	Bovine pericardium (e.g., XenoSure® Biologic patch, LeMaitre Vascular, USA) (diameters: e.g., 1 × 6 cm; 2 × 9 cm) [45]	Polyethylenterephthalat (PTE) patch (Dacron®) [46]	Polyethylenterephthalat (PTE) grafts (Dacron®)
**Portal vein**	10–15 mm	- Peritoneum[47] - Inferior vena cava - Greater saphenous vein - Gonadal vein [35, 39]	- Internal jugular vein - Left renal vein - Splenic vein - Iliac vein [35] - Portal vein/hepatic vein from contralateral liver lobe [48] *Manufactured Neo-tube* - Greater saphenous vein - Peritoneum [47, 49]	Cryopreserved Iliac vein [43, 44]	Bovine pericardium (e.g., XenoSure® Biologic patch, LeMaitre Vascular, USA) (diameters: e.g., 1 × 6 cm; 2 × 9 cm) [45]	- Polytetrafluoroethylene (PTFE; GoreTex®; Gore, USA) - Polyethylenterephthalat (PTE) patch (Dacron®) [2]	-Ringed/not-ringed polytetrafluoroethylene (PTFE) grafts (GoreTex®; Gore, USA) - Polyethylenterephthalat (PTE) grafts (Dacron®) [2]
**Hepatic vein**	8–12 mm	- Peritoneum - Inferior vena cava - Greater saphenous vein - Gonadal vein [50] - Peritoneum [47, 49]	- Internal jugular vein - Left renal vein - Splenic vein - Iliac vein [50] - *Portal vein/hepatic vein from contralateral liver* [48] *lobe*	Cryopreserved Iliac vein [43, 44]	Bovine pericardium (e.g., XenoSure® Biologic patch, LeMaitre Vascular, USA) (diameters: e.g., 1 × 6 cm; 2 × 9 cm) [45]	- Polytetrafluoroethylene (PTFE) grafts (GoreTex®; Gore, USA) - Polyethylenterephthalat (PTE) grafts (Dacron®) [51]	- Ringed polytetrafluoroethylene (PTFE) grafts (GoreTex®; Gore, USA) polyethylenterephthalat (PTE) grafts (Dacron®)[52]
**Inferior vena cava**	20–22 mm	Peritoneum [47, 49]	*Manufactured Neo-tube* Peritoneum [47, 49]	Cryopreserved Iliac vein [43, 44]	Bovine pericardium (e.g., XenoSure® Biologic patch, LeMaitre Vascular, USA) (diameters: e.g., 1 × 6 cm; 2 × 9 cm) [45]	- Polytetrafluoroethylene (PTFE) patch (GoreTex®; Gore, USA) - Polyethylenterephthalat (PTE) patch (Dacron®) [51]	- Ringed polytetrafluoroethylene (PTFE) grafts (GoreTex®; Gore, USA) [52]
**Advantages of the available material for vascular reconstruction**		- Low infectious risk - Low costs - No need for anticoagulation for venous reconstruction		- Low infectious risk - No time-consuming preparation	- Availability - No time-consuming preparation	- Broad availability in form and sizes - No time-consuming preparation - Mechanical stability (ringed grafts)	
**Disadvantages of the available materiasl for vascular reconstruction**		- Size mismatch - Time-consuming preparation - Time-consuming manufacturing in case of neo-tube graft - Extended operative trauma - Less mechanical stability		- ABO incompability contradict - Difficult access	- Limited in size - Time-consuming manufacturing in case of neo-tube graft	- Need of therapeutic Anticoagulation - Risk of thrombotic complications - Risk of infections - High costs - Available > 6 mm diameter	

The harvesting of the portal vein or the hepatic vein from the portion of the liver that will be resected is another option for the use of autologous veins. The advantages of this method are the similar character of the graft and the avoiding of additional operative trauma by using autogenous veins. The disadvantage is that vessels are often not available due to tumor infiltration. Moreover, vessels need to be assessed before removal of the liver, which is technically demanding [48]. However, most surgeons use the greater saphenous vein or the jugular vein as autologous material for reconstruction.

It is important to keep in mind that autologous venous grafts with venous valves need to be inserted in the correct flow direction to avoid primary graft failure.

Autologous arterial grafts for reconstruction used in liver surgery are the splenic artery, the inferior mesenteric artery, or the radial artery [38, 39].

Based on the experience in liver transplantation, cryopreserved arterial or venous homografts from “Homograft Banks” can be also used for reconstruction [43]. In Germany, the German Society for Tissue Transplantation (DSO-G) coordinates and supplies the hospitals with safe homografts [53].

Arterial or venous homografts were first used for vascular reconstruction in liver transplantation [54, 55]. During the last two decades, cryopreserved arterial homografts were also used in vascular surgery for the reconstruction of abdominal aortic infections in non-transplant patients with excellent short- and long-term results [56]. According to the literature, no immunosuppression is necessary. Indeed, many authors do not even require ABO blood group compatibility as cryopreserved homografts seem to lack an endothelial layer (lack CD31) [57]. However, the risk of early homograft degeneration related to ABO incompatibility is controversial [43].

Results reported in the literature are encouraging with excellent short-term graft and patients survival up to 100%. Long-term survival is mainly depending on the underlying disease indicating the graft. After the use of cryopreserved iliac arteries for vascular reconstruction in liver transplantation, 5-year survival rates between 70 and 90% are reported [43, 44, 58].

Besides autologous grafts or homografts, xenogenous material such as bovine pericardium (e.g., XenoSure® Biologic patch, LeMaitre Vascular, USA) can be used for vascular reconstruction [45]. The variety of synthetic grafts is large. The synthetic grafts that are mostly used for vascular reconstruction in liver surgery are polyethylenterephthalat (PTE) grafts (Dacron®) and (ringed) polytetrafluoroethylene (PTFE) grafts (Gore-Tex®, Gore, USA).

Table 1 gives an overview of possible grafts and patches for vascular reconstruction in liver surgery.

## Reconstruction of the hepatic inflow

### Portal vein resection and reconstruction (PVR)

#### General aspects

Portal vein resection in liver surgery is mainly necessary for malignancies located in or close to the hilum of the liver such as perihilar cholangiocarcinoma (Klatskin tumors) or gall bladder carcinomas. However, centrally located metastases or HCC are less frequent, but good indications for portal vein resection as well.

The first reported combined liver and portal vein resection (PVR) with primary anastomosis between the upstream side of the PV and the inferior vena cava was performed successfully for cholangiocarcinoma by Professor Kajitani 1965 in Tokyo [17]. Later, in 1990, Hadjis et al. [46] were the first in the Western hemisphere to report on portal vein resection (PVR) with porto-portal reconstruction in hepatic surgery. Based on experiences of PVR and extended right hemihepatectomy by Klempnauer et al. [59], Neuhaus and colleagues [6] developed in 1999 a no-touch, “en-bloc” technique for right-sided hilar cholangiocarcinoma with standardized portal vein resection and reconstruction to minimize tumor dissemination and to improve local radicality.

As mentioned above, exact knowledge of the anatomy of the PV and its branches is essential for resection planning and should be assessed by imaging before surgery. Figure 2 shows the most common variations in portal vein anatomy according to the classification proposed by Cheng et al. [33] and Nakamura et al. [32].

Portal vein anastomosis after hilar bile duct resections without liver parenchyma can be performed as primary end-to-end anastomosis. After left hemihepatectomy with portal vein resection, portal vein anastomosis is performed with the main portal vein and the right portal vein or with the main portal vein and the right posterior portal vein for extended left hemihepatectomy. For right hemihepatectomy or extended right hemihepatectomy, the main portal vein is anastomosed with the left portal vein prior to branches to segments 2 and 4. Technically, resection/reconstruction of the left portal vein is easier because of its long extrahepatic course compared to the right side.

Transection of the liver parenchyma can be performed before or after PVR. In our own experience, it is recommended to perform this anastomosis prior to hepatic transection, in order to achieve optimal alignment and avoidance of rotation. However, the chronology of surgical steps has to be adapted from case to case depending on the best access. Mekeel and Hemming AW [39], Hemming et al. [60], and Hemming et al. [61] describe the advantages of parenchymal transection prior to vascular reconstruction in the case of left or extended left hemihepatectomy.

For portal vein reconstruction, primary end-to-end anastomosis is recommended if technically feasible.

If primary anastomosis is not possible, autologous grafts, homografts, xenografts, or synthetic material are applied for reconstruction (Table 1). Possible veins for autologous grafts are the left renal vein (preserving the drainage of the left kidney by conservation of either the left suprarenal vein or the left gonadal vein), the splenic vein, or internal jugular vein. Some authors describe the use of the iliac vein [35, 39]. Moreover, the use of the hepatic vein or the portal vein from the part of the liver that will be removed is described in case of tumor clearance in this area. The greater saphenous vein is generally not used as a complete segmental graft due to its small diameter.

However, the creation of a tube by longitudinal incision of the greater saphenous vein and tube-like reconstruction over a syringe can be used for segmental portal vein reconstruction. According to this technique, a tube can also be reconstructed by peritoneum. Noteworthy, the peritoneal layer needs to be on the luminal side of the new tube. The tubes can be manufactured either by a running suture using a monofile, non-resorbable suture (e.g., Prolene® 5–0) or a vascular linear cutting stapler [49].

Synthetic material is usually avoided because of the risk of infection and thromboses, but when needed, a 10 mm PTFE graft (e.g., GoreTex®) or PTE graft (Dacron®) can be interpositioned [2]*.*

Portal wall defects should be reconstructed by autologous patches, if tangential resection of the portal vein is required. Peritoneum/falciform ligament, inferior vena cava, greater saphenous vein, and external/internal jugular veins are used for patch reconstruction of portal vein defects. If the autologous venous patch is not available, bovine pericardium patches can be used as well (Table 1).

In our center, we prefer the use of peritoneal patches or patches of the vena cava for portal vein reconstruction after tangential resection and internal jugular or left renal vein for graft interposition.

#### Surgical technique

The portal vein should be fully mobilized before resection/reconstruction to prevent tension on the suture. The liver should be posed in its final position to avoid rotation of the anastomosis.

End-to-end anastomosis is performed with two running sutures of the back wall and front wall with a monofile, non-resorbable suture (e.g., Prolene® 5–0). It is important just to adapt the suture and knots are slightly slack to create a so-called growth factor to avoid stricture of the vein. Before final closure of the anastomosis and before the blood flow is restored, retro- and antegrade flushing and local application of a solution of heparin/saline (5000 IE heparin/500 ml saline) are performed.

An oblique cut can be helpful to minimize the discrepancy of caliber between the main portal vein and the portal venous branches.

Anastomoses of graft interposition are performed analogous: the distal (liver distant) anastomosis should be performed first. After completion of the distant anastomosis, the distant vascular clamp should be positioned behind the new anastomosis on the graft. By this technique, the sufficiency of the first anastomosis is confirmed avoiding to potentially deal with two insufficient anastomoses at the same time afterwards.

**Fig. 3:**
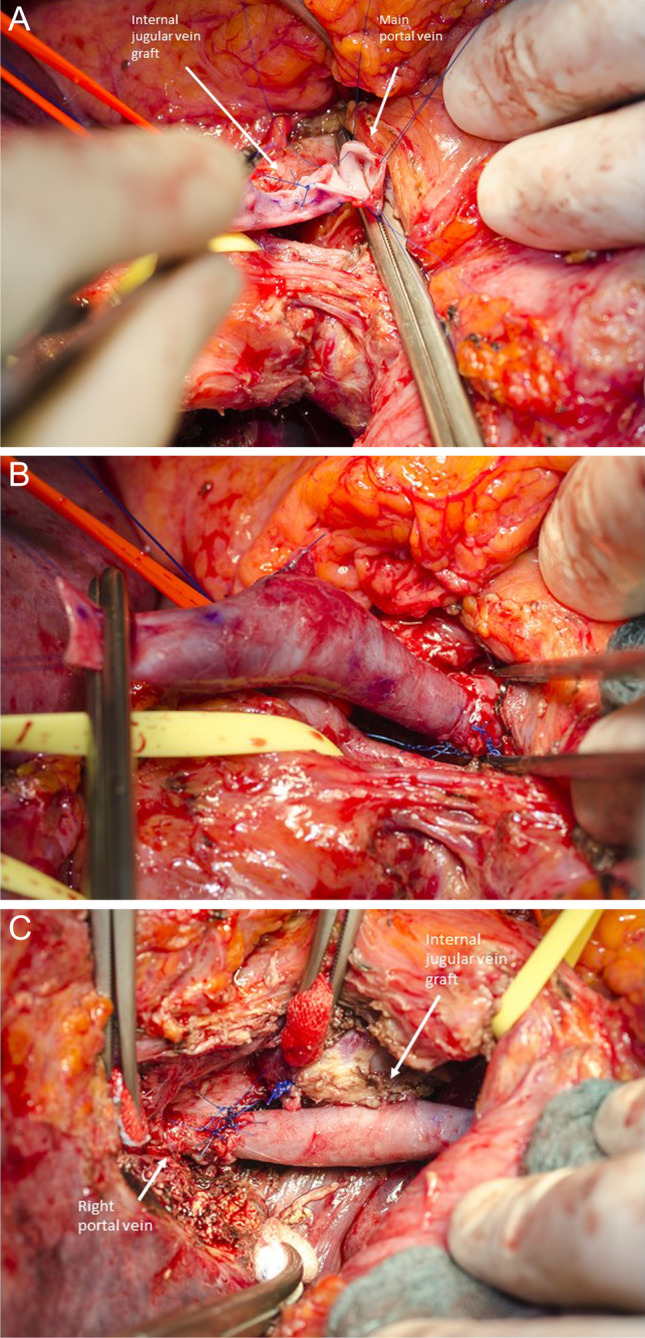
Portal vein reconstruction by the use of the left internal jugular vein as interposition graft in a patient receiving left hemihepatectomy with portal vein resection for cholangiocarcinoma. **a** Distal anastomosis between the main portal vein and the left internal jugular vein graft. On the surface of the jugular vein, correct flow direction is marked. **b** After completion of the distant anastomosis, the distant vascular clamp is positioned more proximally on the graft, proofing sufficient anastomosis. **c** Final result of the portal vein reconstruction after completion of the proximal anastomosis between internal jugular vein graft and the right portal vein

Figure 3 demonstrates portal vein reconstruction by the use of the left internal jugular vein as an interposition graft in a patient receiving left hemihepatectomy with portal vein resection for cholangiocarcinoma (Fig. 3).

#### Oncological aspects and postoperative results

Results from high-volume centers were encouraging for combined PVR in liver resection and this technique became the most frequent applied vascular resection in biliary surgery [62].

Extended hepatectomy with portal vein resection is now performed routinely in high-volume institutions [12, 16].

As mentioned above, the main indications for portal vein resection and reconstruction in liver surgery are hilar cholangiocarcinomas or gall bladder carcinomas. However, PVR can also be necessary for centrally located colorectal liver metastases or hepatocellular carcinomas.

Perioperative results are related to the extent of liver resection as well as graft function. However, long-term results are strongly related to tumor biology.

First experiences in combined liver resection and PVR for cholangiocarcinoma reported mortality rates between 8 and 33% [63–66]. Mortality was high in the group with combined liver, bile duct, portal vein, and arterial resection. The main causes of postoperative deaths were portal vein thrombosis and liver failure. Since then, surgical outcomes improved in high-volume centers due to the experience in transplantation. In more recent studies from the last two decades, mortality rates range from 0 to 5% in liver resection and PVR for hilar cholangiocarcinoma which is equivalent to major hepatectomies without vascular reconstruction [60, 63, 67–70].

Lee et al. [70] and Nagino et al. [67] showed measurable improvements in postoperative results over time due to greater experience in combined hepatectomies with PVR: Lee et al. [70] reported mortality rates of 0% from 2005 to 2008 compared to 9.8% for patients resected between 1989 and 2005 for the same surgical procedure. Similarly, Nagino et al. [67] showed improved mortality rates from 9.6% in 2003 to 2% in 2010. The improved outcome seems to be multifactorial—the implication of PVE and biliary drainage preoperative along with increasing surgical experience are possible factors for better results. Hemming et al. [60] published their results in 2011 showing a tendency towards decreased mortality of 5% (previous series 9%). The outcome was attributed to improved selection of patients, greater surgical experience, the use of PVE, and initiation of standardized biliary drainage of the FLR [67, 70].

However, extended liver resections for hilar cholangiocarcinoma show constantly high postoperative morbidity ranging from 40 to 100%. Noteworthy, that postoperative morbidity after major hepatectomy for hilar cholangiocarcinoma is not related to PVR [63, 71]. The main complications are postoperative liver failure and biliary complications as well as infectious complications. Good postoperative short-term results are only possible with successful portal vein reconstruction. Indeed, the patency of portal vein reconstructions varies between 80 and 100% (Table 2).

**Table 2:** Studies reporting about liver resection with portal vein resection and reconstruction (PVR); *RH right hemihepatectomy; ERH extended right hemihepatectomy; LH left hemihepatectomy; ERH extended left hemihepatectomy; SV great saphenous vein; EIV external iliac vein; CCC cholangiocarcinoma; iCC intrahepatic cholangiocarcinoma; pCCC perihilar cholangiocarcinoma*

Author	Year	Number of cases/indication	Mode of resection	PVR (n)	Technique of vascular reconstruction	In- hospital morbidity %	In-hospital mortality %	Survival rates
Song, Lee et al. [70]	1989–1997	pCCC: *n* = 111	*n* = 56 RH *n* = 15 ERH *n* = 26 EL *n* = 14 other resections	29	- e/e *n* = 17 autologous vein graft (EIV) *n* = 6 - Wedge resection *n* = 6 saphenous vein *n* = 1 bovine patch *n* = 1 primary closure *n* = 4	21	9.8	1-year 91% 5-year 22%
Lee et al. l[70]	2010	CCC: *n* = 366 pCCC: *n* = 302 iCCC: *n* = 64	*n* = 34 bile duct resection *n* = 176 right hemihepatectomy *n* = 11 right trisectionectomy *n* = 121left hemihepatectomy *n* = 9 left trisectionectomy *n* = 4 central bisectionectomy *n* = 11other hepatectomies	38	- e/e *n* = 29 vein graft interposition (LRV or EIV) *n* = 5 - Wedge resection *n* = 4 primary closure *n* = 3 saphenous vein *n* = 1	43	1.7	1-year 85% 5-year 47%
Ebata, Nagino et al. [65]	2003	CCC: *n* = 52	*n* = 10 ERH *n* = 21 RH *n* = 5 ELH *n* = 14 LH *n* = 2 other hepatectomies	52	- Wedge *n* = 20 patch *n* = 2 direct closure *n* = 18 - e/e *n* = 29 intreposition graft *n* = 3 (EIV)	27	9.6	3-year 26% 5-year 10%
Nagino et al. [67]	2010	CCC: *n* = 50	*n* = 26 ELH *n* = 23 LH *n* = 1 RH	50	- e/e *n* = 34 - Wedge *n* = 3 *n* = 2 patch(SV) *n* = 1 direct closure -Graft interposition *n* = 13 (EIV)	54	2	1-year 79% 5-year 30%
Hemming et al. [72]	2006	CCC: *n* = 60	*n* = 12 ELH *n* = 37 ERH *n* = 8 LH *n* = 2 RH	26	NA	40	8	5-year 39%
Hemming et al. [60]	2011	CCC: *n* = 95	*n* = 21 ELH *n* = 63 ERH *n* = 8 LH *n* = 3 RH	42	e/e *n* = 42	36	5	5-year 43%
Miyazaki et al. [63]	2007	CCC: *n* = 161	*n* = 59 ELH *n* = 57 ERH/RH *N* = 24 other resections *N* = 20 hilar bile duct resection	41	- 39 e/e - 2 autologous vein graft	39	7	1-year 31% 5-year 17%

Regarding the oncological aspect of extended liver surgery, the implementation of vascular resection increases the number of resectable tumors and cure rates.

For hilar cholangiocarcinoma, en bloc tumor resections in “no touch technique” as advocated by Neuhaus et al. [73] in 1999 showed significantly improved 5-year survival rates of 58% after resection for cholangiocarcinoma. Other studies reported that microscopic invasion of the portal vein doesn’t influence the prognosis, but patients who underwent portal vein resection had worse survival [65]. Due to higher technical demand and a lack of broad validation of this technique, many centers perform portal vein resection only in case of macroscopic infiltration of the portal vein [74, 75].

Two authors published a multivariate analysis, presenting that the prognosis in patients with PVR was worse than in the group without PVR [65, 66].

Comparably less literature is available regarding oncological outcomes after major hepatic resections with PVR for HCC or CRLM. According to our own experience, major hepatectomies with PVR have favorable results for those indications.

#### Excursion: thrombectomy for HCC tumor thrombus in the portal vein

**Fig. 4:**
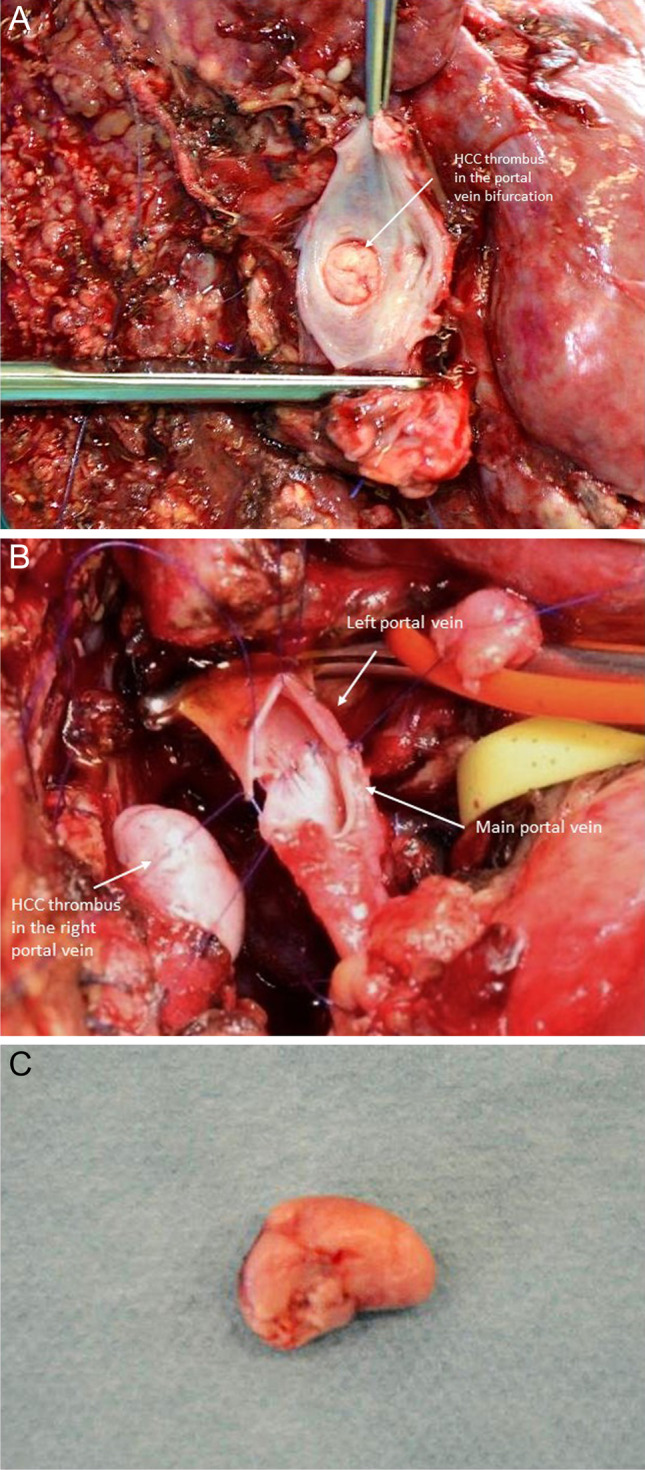
Patient with hepatocellular carcinoma (HCC) and HCC tumor thrombus in the portal vein bifurcation receiving right hemihepatectomy with portal vein resection, intravascular HCC-tumor thrombus evacuation, and end-to-end anastomosis of the main portal vein and the left portal vein. **a** Right hemihepatectomy specimen with tumor thrombus in the portal vein bifurcation after right hemihepatectomy with open resection of the portal vein bifurcation. **b** Portal vein resection and reconstruction with a primary end-to-end anastomosis. **c** Evacuated HCC tumor thrombus

Tumor thrombus in the portal vein is sometimes seen in patients with HCC (Fig. 4a). Although these patients were considered to have dismal prognosis and are no standard candidates for surgery according to the BCLC (Barcelona Clinic Liver Cancer) algorithm, several studies showed improved survival and quality of life for those patients after surgery compared to chemotherapy or best supportive care (BSC) [76]. Fan et al. showed a 5-year survival rate of 22.4% after surgery for patients with portal vein tumor thrombus compared to 0% in the group with conservative treatment [77]. Another study compared a similar cohort of patients with HCC and portal vein tumor thrombus for results after hepatic resection and transarterial chemoembolization (TACE) with significantly better survival in the surgery group (11.1 vs. 0.5%). A new approach might be to radiate the tumor thrombus in the portal vein preoperatively [78].

#### Surgical technique

For evacuation of HCC tumor thrombus from the portal vein, open resection of the portal vein bifurcation is necessary. HCC tumor thrombi are mostly not adherent to the venous wall and can easily be removed by pulling out of the vein. Primary closure of the portal vein without resection is sufficient for reconstruction in those cases. Figure 4 shows the case of patients with a huge HCC of the right liver lobe with HCC tumor thrombus in the portal vein bifurcation reaching into the left portal vein. In this case, right hemihepatectomy with “open access” and incision of the portal vein bifurcation by resection of the right portal branch under vascular exclusion of the left portal vein and the main portal vein was performed. The HCC tumor thrombus was not adherent to the wall and could be easily evacuated. The portal vein was reconstructed by primary closure of the portal vein bifurcation by a running suture using Prolene® 5–0 (Fig. 4).

However, some HCC thrombi are adherent to the venous wall; in those cases, removal of the thrombus by partial wall resection or segmental resection of the portal vein is necessary.

### Hepatic artery resection and reconstruction

#### General aspects

Indication for resection and reconstruction of the hepatic artery in liver surgery is similar to the PVR. Mainly, pCCC or gall bladder carcinomas are infiltrating the proper hepatic artery or/and its main branches. Frequently, additional infiltration of the portal vein is present and requires simultaneous portal vein resection and reconstruction.

The broad experience with hepatic artery resection and reconstruction has been gained from liver transplantation (especially living-donor liver transplantation) and surgery of perihilar cholangiocarcinoma [79]. Initial reports of arterial reconstruction within oncological major liver resections were not encouraging because of anastomotic problems (stenosis, occlusion) and unfavorable oncological long-term results. Technical success of arterial reconstruction in pancreatic surgery led to more aggressive resections in hepatic malignancies, in order to achieve negative margins and to increase resectability rates [39]. These procedures are still controversially discussed with regard to their complexity, and contradict results in the literature.

**Fig. 5:**
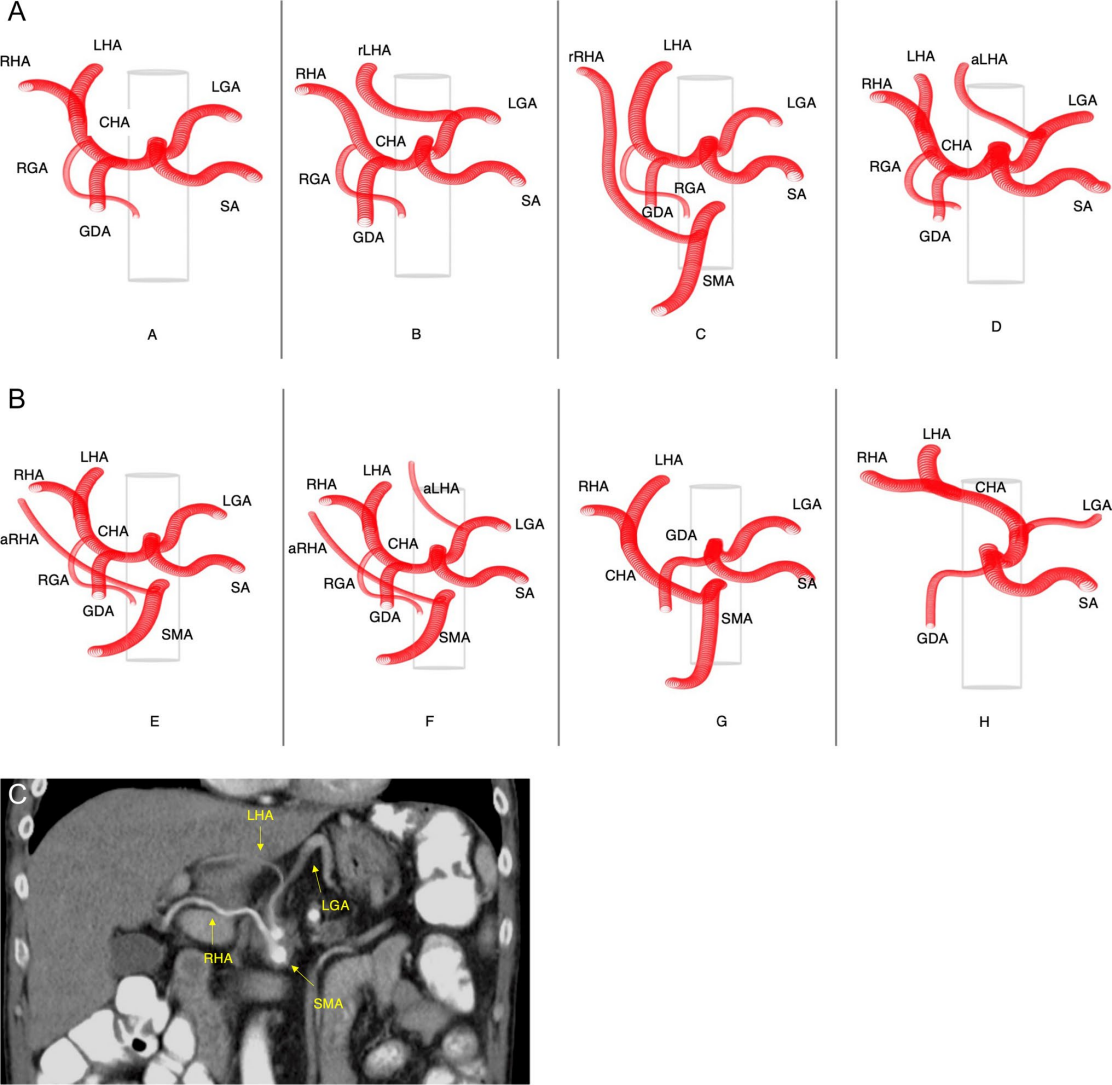
**a** and **b** Anatomy of the hepatic artery/coeliac trunk and common anatomical variants (modified Michels classification). A: normal anatomy (common variant); common hepatic artery (CHA) originating from the coeliac trunk (with left gastric artery (LGA) and splenic artery (SA)), then dividing into left and right hepatic artery after release of gastroduodenal (GDA) and right gastric artery (RGA). B: replaced left hepatic artery (rLHA) originating from the LGA. C: replaced right hepatic artery (rRHA) originating from the superior mesenteric artery (SMA). D: accessory left hepatic artery (aLHA) originating from the LGA. E: accessory right hepatic artery (aRHA) originating from the SMA. F: aRHA and aLHA originating respective from SMA and LGA. G: CHA originating from SMA. H: CHA originating from LGA. **c** Patient’s computed tomography (CT) showing an anatomical variation with a combination of RHA originating from the SMA and the LHA originating from the LGA. *Common hepatic artery* = *CHA, splenic artery* = *SA, superior mesenteric artery* = *SMA, right hepatic artery* = *RHA*

Good preoperative vascular imaging is important to plan the arterial resection and assess the options for reconstruction. Similar to PVR, detailed knowledge of anatomy is mandatory for performing vascular resection and avoiding damage of the arterial supply to the liver. A regular hepatic arterial anatomy is being found only in 55% of the population and thus by anticipating the anatomic variants the risk of hepatic arterial complications can be reduced [8]. Figure 5 gives an overview of the most common anatomic variations of hepatic arteries according to the Michels classification published 1966 [80].

It is important that the proximal and distal sites of the artery are clear of tumor after resection.

Resection and reconstruction can be performed before or after the transection of the liver. Many authors prefer arterial resection and reconstruction prior to liver transection to preserve arterial blood flow to minimize liver ischemia and avoid traction and disruption of the anastomosis. Again, the chronology of surgical steps is always depending on good access to the artery and has to be decided according to the individual case.

Anastomoses of the common hepatic artery, the common hepatic artery with the left hepatic artery, or the common hepatic artery with the right hepatic artery are generally performed. Anastomoses to sectoral branches such as the right posterior hepatic artery after extended left hemihepatectomy or the segment 2/3 artery after extended right hemihepatectomy are possible, but technically much more demanding and associated with a higher risk.

End-to-end reconstruction is performed, if possible. The use of interposition grafts (saphenous vein, gonadal vein, splenic artery, inferior mesenteric artery, radial artery) or transposition of another artery (left gastric artery, gastroduodenal artery; see below) is often necessary if no tension-free end-to-end anastomosis is feasible [38, 39].

Recently, the use of arterial grafts seems to be advantageous for arterial reconstruction due to structure, diameter, and patency. In small patient series, some encouraging results were reported for the use of autologous inferior mesenteric artery in the reconstruction of the hepatic artery [40, 41]. Alternative inflow such as the gastroduodenal artery or left hepatic artery anastomosed with the posterior branch of the right hepatic artery may also be used for extended left hepatectomy [81]*.*

Revascularisation with synthetic grafts should be avoided due to the small size of the vessels and the risk of infection or thrombosis. However, in very rare instances an iliaco-hepatic or aorto-hepatic synthetic interposition graft is necessary.

#### Surgical technique

The artery must be fully mobilized before reconstruction to prevent tension on the suture. Before clamping of the hepatic artery, 3000 IE of unfractioned heparin i.v. should be administered systemically. Special care should be taken when clamping the vessel to avoid intimal tears.

For end-to-end anastomosis, a running suture of the posterior wall and interrupted stitches of the ventral wall or a reconstruction with interrupted stitches in very small arteries with a monofile, non-resorbable suture (e.g., Prolene® 6–0) is the preferred technique. Before the final closure of the anastomosis and restoration of the blood flow, retro- and antegrade flushing and local application of a solution of heparine/sodiumchloride (5000 IE heparin/500 ml saline) are performed.

Anastomoses of autologous graft interposition are performed analogously. Noteworthy, in the case of using venous grafts such as the greater saphenous vein, flow direction should be marked on the surface immediately after preparation to avoid insertion in the wrong direction to avoid perfusion disturbances. This is true for all venous interposition grafts with venous valves.

The distal (liver distant) anastomosis should be performed first. After completion of the distant anastomosis, the distant vascular clamp should be positioned behind the new anastomosis on the graft. By this technique, the sufficiency of the first anastomosis is confirmed avoiding to potentially deal with two insufficient anastomoses at the same time.

The use of magnifying glasses is considered mandatory by most surgeons for the reconstruction of small arteries to avoid dissection of the intima. Some surgeons even prefer to use a surgical microscope for arterial anastomosis with excellent results [67].

To our own experience, good results of arterial anastomoses are obtained by using magnifying glasses.

#### Oncological aspects and postoperative results

Reports about arterial reconstruction in oncological liver surgery are almost exclusively including patients with hilar cholangicarcinoma or—rarely—gall bladder carcinoma.

In most series, arterial reconstruction is performed combined with portal vein reconstruction.

Data of early series with arterial resection from 1980 to 2009 for cholangiocarcinoma were not encouraging with high morbidity rates up to 80 [63, 82]% and mortality rates up to 55% [63, 82]. Indeed, in an analysis by Miyazaki et al. [63], morbidity increased significantly after arterial reconstruction in liver resections for hilar cholangiocarcinoma from 36 to 78%. In another study about hepatectomies for hilar cholangiocarcinoma, multivariate analysis identified arterial reconstruction as a significant factor for postoperative mortality [83]. In 2013, Abbas et al. [84] published a meta-analysis including 24 studies and demonstrated an increased morbidity and mortality for resection of hilar cholangiocarcinoma with arterial reconstruction, but without a survival benefit. Different from those results, other authors didn’t report increased morbidity and mortality for combined arterial and portal vein resection/reconstruction in hilar cholangiocarcinoma [39, 70, 85]. The largest series was reported by Nagino et al. [67], including 50 patients with combined arterial and portal venous reconstruction in hepatectomies for hilar cholangiocarcinoma. One-year and 5-year survival rates of 78.9% and 30.3% were reported. Noteworthy, complete R0 resection was only achieved in 60% of patients. Some of the arterial anastomoses were performed by the use of microsurgical techniques [67]. Similar good results were reported by Yamanaka: out of 25 major hepatic resections for hilar cholangiocarcinoma,10 patients received arterial reconstruction. Eighty percent of arterial anastomoses were performed under microsurgical techniques. Postoperative mortality was reported at 8.8% [64].

Besides the use of microsurgical technique, experience from the living-donor liver transplantation had a major impact on improved techniques in arterial resection and reconstruction for hepatectomies in high-volume centers. Especially during the last decade, acceptable outcome was reported. Berumen and Hemming [86] reported in 2016 on 4% perioperative mortality after arterial resection in a mixed cohort of patients with CCC and gallbladder carcinoma presuming that improved surgical techniques and experience may have improved short-term outcomes.

Due to missing other curative treatment options and fatal courses under palliative chemotherapy, surgery with arterial reconstruction should be generally considered if technically feasible.

#### Portal vein arterialization

If arterial anastomosis is not feasible for technical reasons, portal vein arterialization (PVA) is described as a salvage procedure [87, 88]. In this case, direct end-to-side anastomosis of the hepatic artery to the portal vein is performed. This procedure can be associated with biliary complications and hemorrhage due to portal hypertension. Nevertheless, in a study with only 16 patients, PVA was reported to be successful in 60% of cases [87]. This technique should be only performed when other options are not available since it carries a substantial risk of complications.

Some surgeons advocate to perform a second operation several weeks later and close the arterio-portal anastomosis after initial arterialization to avoid long-term complications of portal hypertension.

In authors’ own experience, no convincing results could be obtained as this procedure causes loss of arterial perfusion and compromises portal venous flow of the liver.

#### Excursion: hepatic artery aneurysm

Different from oncological indications, aneurysm of the hepatic artery is another indication for resections and reconstruction of the hepatic artery. Hepatic artery aneurysm represents nearly 20% of all visceral artery aneurysms and is mostly located in the common hepatic artery (CHA). Surgical or endovascular treatment is available and indicated for aneurysms with a diameter ≥ 2 cm due to the high risk (up to 80%) of rupture [89, 90]. Although both techniques are effective and no significant difference in mortality has been reported, endovascular treatment is less invasive and the preferred alternative to surgery by some authors [91]. The surgical approach contains the excision of the aneurysm sac and revascularization with direct end-to-end reconstruction or interposition of venous or prosthetic graft as described above. The endovascular alternatives are endograft exclusion or coil embolization. The last one is highly selective and associated with less hepatic ischemia especially in cases of intrahepatic aneurysms [92].

The decision for endovascular or surgical procedure depends on the location of the aneurysm, the clinical status of the patient, and the presence of collateral arteries.

However, hepatic artery aneurysm is mostly treated by vascular surgeons and is generally not a domain of liver surgeons in most centers.

## Reconstruction of the vascular hepatic outflow

### Resection and reconstruction of the hepatic veins

#### General aspects

Maintaining a suitable blood outflow is as essential as maintaining a sufficient blood inflow in liver surgery. Preservation or reconstruction of one adequately draining hepatic vein is sufficient to maintain liver function [93]. Resection and reconstruction of hepatic veins (HV) are technically much more demanding due to their short extrahepatic course and localization compared to inflow reconstruction. In addition, hepatic veins have a high risk of kinking because of low intraluminal pressure and the change of position of the remnant liver while undergoing postoperative hypertrophy.

Infiltration of hepatic veins is often combined with infiltration of the inferior vena cava (IVC). Therefore, hepatic vein resection is often performed in combination with vena cava resection. Resection and reconstruction of hepatic veins requires often total vascular exclusion of the liver and might be performed under in situ cold perfusion, ante situm resection, or ex situ resection as described below *in section IVC.*

Due to the increasing experience and development of surgical techniques and approaches—mainly in liver transplantation—even complex reconstruction of the hepatic veins is nowadays possible.

Similar to other vascular resections in liver surgery, a good preoperative imaging combined with detailed knowledge of anatomy is essential for planning the resection with avoiding venous congestion and outflow impairment [79]. Recent publications report on the potential advantage of preoperative planning based on three-dimensional visualization of the liver [94, 95]. Important anatomic variations are seen regarding the confluence of the left and middle hepatic vein (MHV) as well as the existence of a strong fissural or a large inferior hepatic vein (20–24% of the patients) [96].

**Fig. 6:**
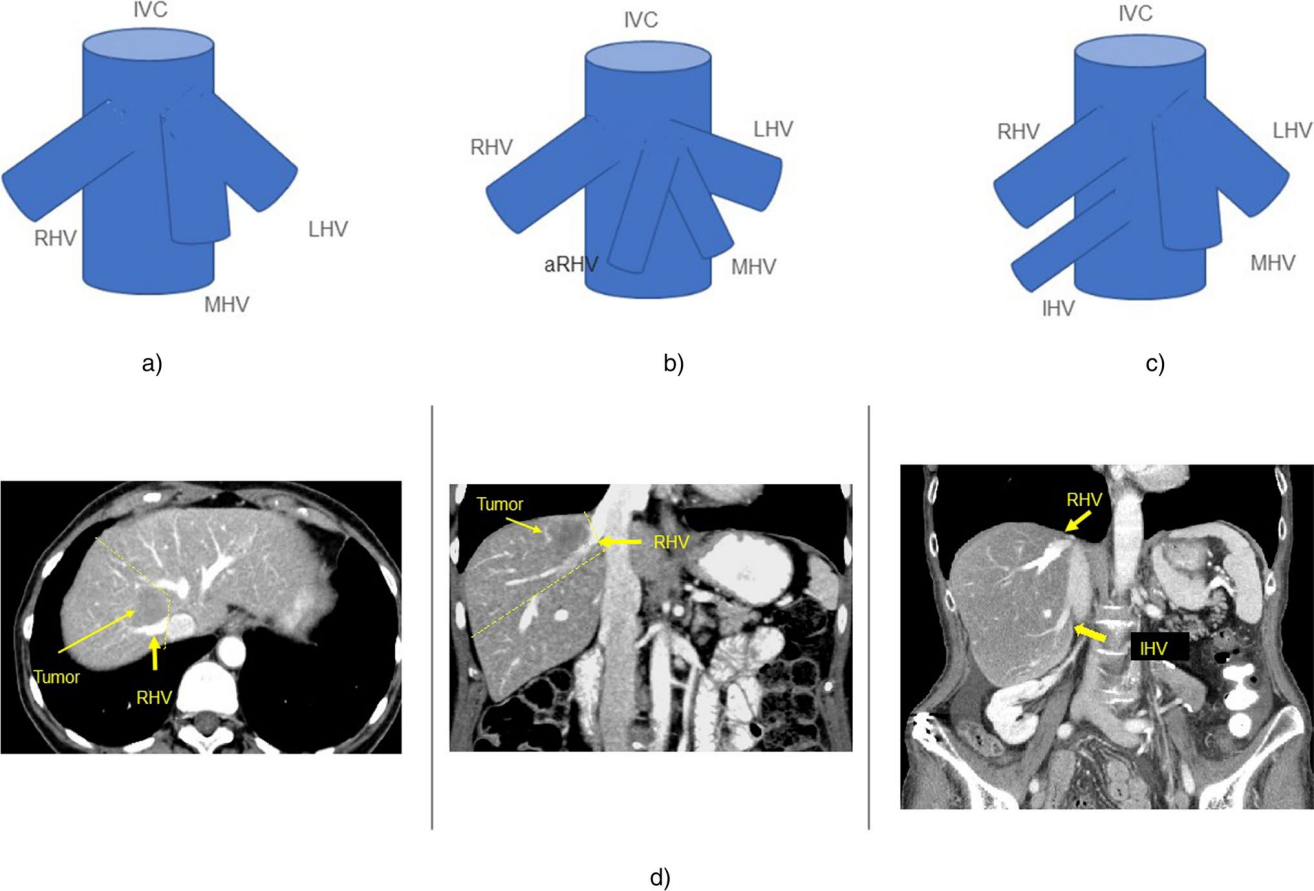
Anatomy of the hepatic veins and common anatomical variants. **a** Normal anatomy (common variant); right hepatic vein (RHV) and common trunk of the middle (MHV) and left hepatic vein (LHV). IVC = inferior vena cava. **b** Accessory right hepatic vein (mostly segment 8 vein) (aRHV) draining in a common trunk with the MHV and LHV. **c** Accessory inferior right hepatic vein (IHV) seen in 47% of cases [8]. **d** CT scan of a patient with a tumor located in liver segments 7/8 and the anatomic variation of an inferior hepatic vein. In this case, resection of segment 7 and 8 with resection of the right hepatic vein without reconstruction was possible. Outflow of the liver segments 5 and 6 via the strong inferior hepatic vein was sufficient. The yellow line encircles the resection area of the tumor with the right hepatic vein

The most important anatomic variations are a prominent inferior hepatic vein, draining segments (mostly segments 6 and 7) of the right side of the liver. Realization of this vein is important to avoid bleeding complications during preparation as well as to support extended possibilities of liver resection including hepatic veins without vascular reconstruction as mentioned above (Fig. 6). Another important venous variant is a branch draining the liver segment 8 into the MHV, which is present in 9% of the patients. In the case of left hemihepatectomy, the resection of the MHV may lead to impairment of the outflow of liver segment 8 [8]. In case of an extended right hemihepatectomy with resection of the middle hepatic vein, if present, a variant drainage of the segment 3 vein in the MHV needs to be reconstructed. Depending on the location, not all hepatic venous variants are relevant for the tumor resection, but good preoperative imaging is important to provide information about the venous drainage. Main anatomic variations are displayed in Fig. 6a–c. CT scan of a patient with a tumor located in liver segments 7/8 and the anatomic variation of an inferior hepatic vein (arrow) is shown in Fig. 6d.

The intraoperative detection of communicating veins between the three major hepatic veins may be helpful to perform parenchyma-sparing resection for tumors involving the major hepatic veins. These conjunctions are reported to be presented in 80% of the patients and can be detected by using an intraoperative ultrasound of the liver [97].

In the case of infiltration of the left, middle, and right hepatic vein with the need for reconstruction of at least one of the veins, the intrahepatic part of the hepatic vein that needs to be reconstructed must have a sufficient size to qualify for reconstruction.

Primary anastomosis of hepatic veins or direct re-implantation into the vena cava is often difficult or impossible because of their short extrahepatic course. However, if feasible, direct re-implantation is always the preferred option as this lowers the risk of kinking of the venous reconstruction.

In most cases, the use of autologous, allogenic, or synthetic patches or interposition grafts is necessary to reconstruct hepatic veins.

#### Surgical technique

**Fig. 7:**
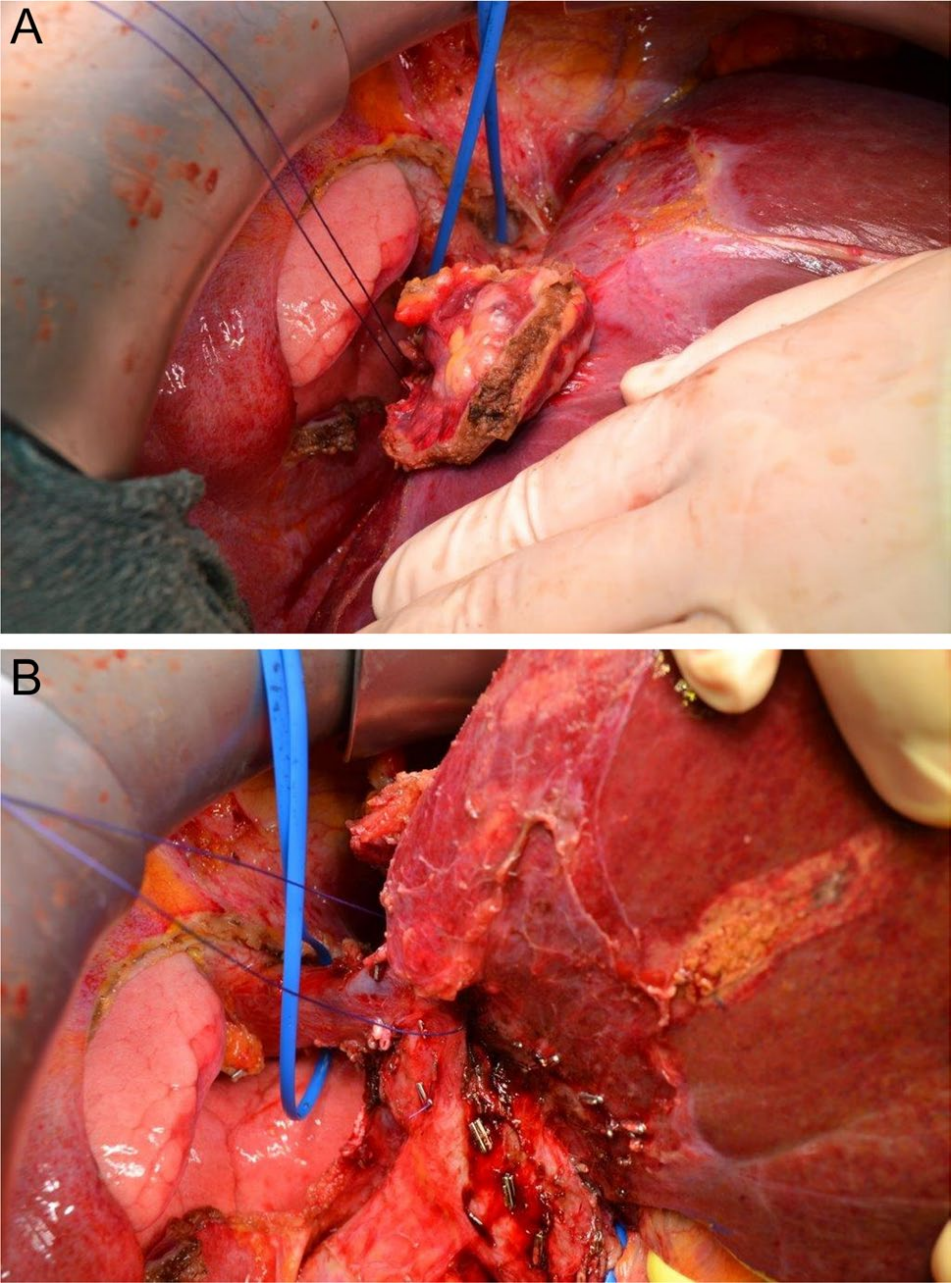
**a** and **b** Patient with a recurrent colorectal liver metastasis (CRLM) of segment 4a/8 infiltrating the diaphragm. En bloc tumor resection with liver and diaphragm was performed without vascular resection. Exzision of the tumor-infiltrated area of the diaphragm has already been performed and enables the view onto the right lung. Tumor is still adjunct to the liver. Vascular control of the suprahepatic vena cava and the right hepatic vein: the blue rubber band encircles the supradiaphragmatic IVC, the blue ligature encircles the right hepatic vein

Before partial or complete resection of a hepatic vein, the IVC above the venous confluence and the IVC below the hepatic venous confluence as well as all hepatic veins need to be mobilized and encircled to have complete bleeding control. If necessary, the diaphragm needs to be incised to encircle the suprahepatic IVC. Figure 7 shows vascular control of the suprahepatic vena cava and right hepatic vein after incision of the diaphragm due to excision of a recurrent CRLM infiltrating the diaphragm. Vascular resection was not necessary in this case. Sometimes, an incision of the pericardium is necessary to control the IVC. Noteworthy, the opening of any hepatic vein does not only cause bleeding complications, but also causes risk for fatal air embolism.

Reconstruction of hepatic veins is mostly performed under total vascular exclusion (TVE) of the liver as described below *in section IVC*.

Defects of partial or tangential resection of HV can be performed by the use of peritoneal patches, patches of the greater saphenous vein, the internal jugular vein or bovine pericardium patches. Primary closure of the wall defect is not possible as it will surely lead to relevant stenosis due to the small diameter of hepatic veins (Table 1).

**Fig. 8:**
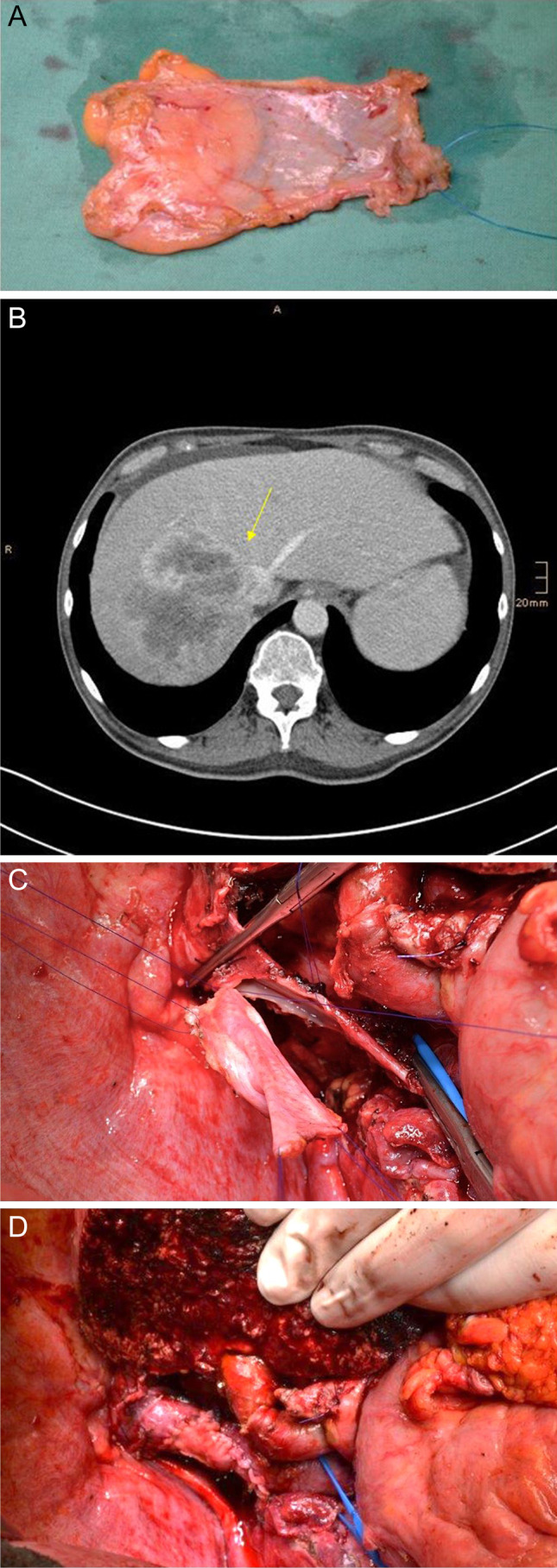
Patient with iCC of the right liver lobe infiltrating the right and middle hepatic vein as well as the IVC. Vena cava reconstruction was performed with peritoneal patch after tangential resection of the vena cava and resection of the middle hepatic vein combined with extended right hemihepatectomy. **a** Patch harvested from the parietal peritoneum. **b** Patient’s CT scan shows the tumor (arrow) infiltrating the IVC, the right and middle hepatic vein. **c** and **d** Reconstruction of vena cava after tangential resection by using the peritoneal patch

In our own experience, we use peritoneal patches or patches from the internal jugular vein for the reconstruction of the wall of the HV after tangential resection. As mentioned above, the grafts should be obtained before vascular exclusion and resection of the hepatic vein**.** Peritoneal patches may be harvested at any part of the parietal peritoneum before resection of the hepatic vein and preserved in a cold heparin/sodium chloride solution. The peritoneal layer is then placed on the intraluminal side of the vessel and a running suture or interrupted stitches using a monofile, non-resorbable suture (e.g., Prolene® 5–0) is performed after fixation of the patch in both angles of the defect. A peritoneal patch is shown in Fig. 8a.

For graft interposition after segmental resection of the HV, internal jugular vein, renal vein, or portal or iliac vein can be used. It is also possible to construct a tubelike interposition graft from the peritoneum or falciform ligament.

As mentioned above, the grafts should be obtained before vascular exclusion and resection of the hepatic vein. Depending on the selected autologous graft, the correct direction of graft interposition has to be controlled as some veins have venous valves. Marking of the flow direction on the graft surface immediately after withdrawal is recommended. Grafts are preserved in a cold heparin/sodium chloride solution. Interposition grafts are inserted by running suture or interrupted stitches using a monofile, non-resorbable suture (e.g., Prolene® 5–0).

Synthetic grafts can be used if no autologous material is available, but should be avoided due to infectious and thrombotic complications as already described in other series [2] (Table 1).

Reconstruction of hepatic veins is often technically very demanding, especially when long intrahepatic preparation of the vein is necessary. Ante situm or ex situ techniques are good options to create better exposure. Those techniques are described hereinafter.

#### Oncological aspects and postoperative results

Different from hilar structures, hepatic veins are often infiltrated by CRLM, HCC, and also iCC or other metatases. Saiura et al. [50] reported a series of 16 patients undergoing hepatic resections with HV reconstruction for CRLM. HV resection and reconstruction were performed with an autologous graft using the great saphenous vein in most cases and the external iliac or portal vein in some patients. If a sleeve resection of the HV was performed, a patch of the umbilical or gonadal vein was used for the reconstruction. No in situ cold perfusion is reported within this series. The authors reported no mortality and 50% perioperative morbidity with only 1 major complication. Long-term survival was 93%, 76%, and 76% after 1, 3, and 5 years respectively and all reconstructed vessels were patents at follow-up [50]. In another cohort reported by Hemming et al. [11], hepatectomies with HV reconstruction for 9 HCC, 5 CRLM, and one CCC were included. Segmental resection and primary reinsertion of the hepatic vein into the IVC or the right hepatic vein were possible in eight patients. In four patients, a Gore®-Tex interposition graft was used and another 4 patients required combined HV and IVC resection and reconstruction. Within the 3-month follow-up, only 2 patients (12%) died of which one patient was deceased 3 months after resection due to an incarcerated diaphragmatic hernia. Although synthetic grafts are at the risk to cause long-term strictures and thrombosis, all vascular reconstructions remained patent in this report. The authors conclude, despite a short median follow-up of 23 months, that late graft complications are probably less important. The 3-year survival rate in this study was 50% [11].

#### Excursion: thrombectomy for HCC tumor thrombus in hepatic veins and/or vena cava

Advanced HCCs tend to infiltrate not only the portal vein, but also the IVC. Although the location of the HCC tumor thrombus in the IVC is related to a worse prognosis, the outcomes after resection of these tumors are acceptable. Especially the quality of life is much better due to the fact that a HCC thrombus in the vena cava extended to the right atrium may cause heart failure, embolism, and sudden death [98]*.* In rare cases of HCC with tumor thrombus in the hepatic vein and in the right atrium, a cardiopulmonary bypass is used in addition to TVE. Those cases have a low incidence of 1–4% and have a very poor prognosis. The procedure is hazardous and demands interdisciplinary collaboration with cardiovascular surgeons [99, 100].

## Resection and reconstruction of the inferior vena cava (IVC)

Tumor infiltration of the inferior vena cava (IVC) can be located distantly to the hepatic veins (HV) or involving the IVC-HV confluence. The type and extent of the IVC resection depend on the location of the tumor. Procedures involving the IVC-HV confluence are technically much more demanding and require different surgical approaches compared to “simple” IVC infiltration.

### IVC resection with preservation of the in- and outflow of the liver

#### Tangential IVC resection distant to the IVC-HV confluence

In case of tangential infiltration of the IVC, tangential resection and closure of the IVC can be performed.

The current series reported an excellent outcome with 97% patency in follow-up after reconstruction with peritoneo-fascial patch grafts during HPB surgical procedures [47, 101]. In our institution, we prefer the use of peritoneal patch harvested from the parietal peritoneum for IVC reconstruction after tangential resection. Figure 8 shows a case of tangential IVC resection reconstructed by the use of a peritoneal patch in a patient with iCC receiving extended right hemihepatectomy (Fig. 8).

##### Surgical technique

Partial resection of the IVC can be either done by a tangential resection using a vascular stapler or by open resection and primary closure of the wall defect by a running suture. Smaller wall defects up to 30% of the circumference of the IVC can be reconstructed by primary anastomosis. However, care should be taken to avoid stenosis.

The defect can be closed primarily by direct suture with a monofile, non-resorbable suture (e.g., Prolene®4/0). For wall invasion larger than 2 cm and involvement up to 30%, a patch can be used to prevent stenosis. If a tumor is involving the IVC more extensively, but less than 60% of its circumference and on a short segment of 2 cm, partial cava clamping is possible to preserve the blood flow in the IVC [102],. Patches can be autologous (venous or peritoneal) or heterologous (bovine) (Table 1). As mentioned before, peritoneal patches have several advantages being easily available, with low risk of infection, low costs, and no need for anticoagulation.

Patches should be obtained before vascular clamping. Liver and IVC should be mobilized from the retroperitoneum to obtain bleeding control. If possible, parenchymal transection should be performed before IVC resection.

**Fig. 9:**
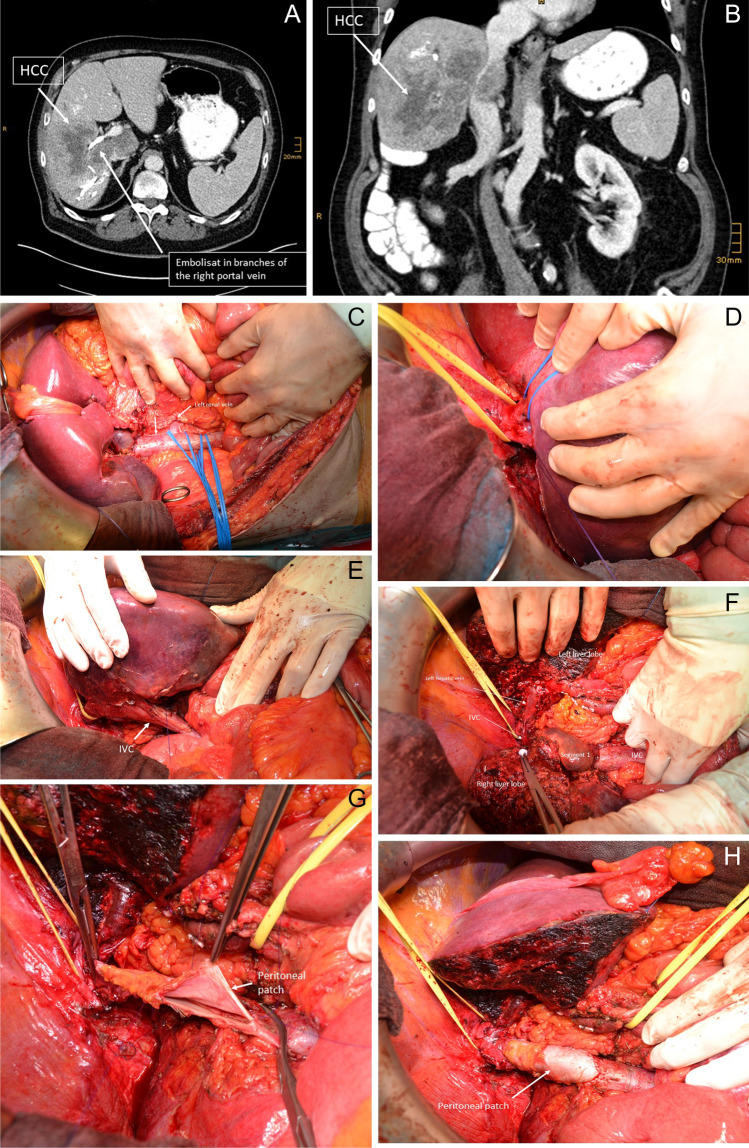
**a**–**h** Patient with HCC of the right liver lobe infiltrating the inferior vena cava (IVC) as well as the right hepatic vein and the middle hepatic vein. Preoperative portal vein embolization of the right portal vein was performed. The left hepatic vein was dissected within parenchyma and preserved. After transection of liver parenchyma, tangential IVC resection was performed to complete tumor resection. **a**–**b** Patient’s CT scan shows the tumor infiltrating the IVC, the right and the middle hepatic vein. **c**–**e** Complete mobilization of the IVC to obtain vascular control: **c** Infrahepatic IVC with blue rubber bands around the infrarenal and suprarenal IVC as well as around the left renal vein. **d** Suprahepatic IVC and liver veins for bleeding control. Yellow rubber band around the suprahepatic IVC; blue rubber band around the left hepatic vein. **e** Complete mobilization of the retrohepatic vein before transection of the liver parenchyma. **f** Complete transection of the liver parenchyma (anterior approach) before partial resection of the IVC. **g** Reconstruction of the ICV by inserting a peritoneal patch harvested from the right abdominal wall. **h** Final result after completed reconstruction of the IVC

Figure 9 shows a case of a patient with a HCC of the right liver lobe infiltrating the inferior vena cava as well as the right liver vein and the branches of the middle hepatic vein. The left vein was dissected within the parenchyma and preserved. After parenchymal transection as an anterior approach for right hemihepatectomy, tangential IVC resection was performed to complete tumor resection. IVC defect was closed by using a peritoneal patch harvested from the right abdominal wall. Surrounding fat tissue and fascia are left on the patches seen in Fig. 9.

#### Segmental resection of the IVC distant to the IVC-HV confluence

In case of more extended tumor infiltration of the vena cava, distant to the IVC-HV confluence, segmental resection of the vena cava needs to be performed. In this case, prosthetic replacement is needed for the reconstruction of the IVC. Synthetic grafts such as PTFE or Dacron grafts can be used as well as autologous material. Some authors recommend using constructed tubes based on parietal peritoneum to avoid synthetic material [49]. However, most authors recommend an 18–20 mm ringed PTFE (Gore-Tex) graft for reconstruction because of its resistance to compression by the liver [103–112].

##### Surgical technique

Chronology of different surgical steps is depending on tumor size and localization. Completed parenchymal transection of the liver as well as resection of the inflow structures of the resected liver with only remaining tumor infiltration on the IVC can be helpful before IVC resection and reconstruction. However, in some cases, the mobilization, resection, and reconstruction of the IVC before completing en bloc tumor resection might be more convenient.

Irrespective of selected chronology, grafts should be obtained before vascular exclusion and resection of the IVC. Complete mobilization of the liver and the IVC should be performed before clamping of the IVC below the venous confluence and above the right renal vein. Administration of i.v. heparin is not mandatory before IVC clamping to avoid bleeding complications from the transection plane of the liver. In our institution, no heparin is administered before IVC clamping; in our own experience, no increased rate of intraoperative thrombotic or thromboembolic complications is observed. However, most authors recommend i.v. heparin before IVC clamping [113].

Resection of the IVC segment is then performed. The defect of the IVC is reconstructed by the use of a ringed synthetic graft (e.g., GoreTex®). The synthetic graft is inserted by using a non-resorbable, monofile running suture (e.g., Prolene® 4/0).

Before final closure of the anastomosis and restoring of the blood flow, retro- and antegrade flushing and local application of a solution of heparin/saline (5000 IE heparin/500 ml saline) are performed.

Reconstruction of the IVC segment can also be performed by inserting a constructed autologous peritoneal tube. The parietal peritoneum is harvested from the ventral or lateral abdominal cavity covered by the posterior rectus sheet. The peritoneal surface has to be on the luminal side of the tube. The peritoneal patch can then be wrapped around a syringe of 20–22 French and a customized tube is constructed by longitudinal anastomosis with a vascular linear cutting stapler. The advantage of this graft is the low risk of infection. However, this reconstruction has less stability compared to a ringed synthetic graft. This might be problematic in larger retrohepatic IVC reconstructions.

#### Caval shift procedure

As mentioned above, biliary leakage is a common complication in liver surgery with a high risk of contamination and infection of synthetic material with fatal consequences. In order to avoid infectious complications of synthetic vascular grafts, a so-called caval shift procedure can be performed.

The basic idea of the caval shift procedure is to create sufficient distance between the liver and the synthetic graft to avoid infection of the synthetic graft. Therefore, an appropriate segment of the infrarenal IVC is resected and preserved and the infrarenal defect of the IVC is reconstructed by a synthetic graft that is distant to the liver and its possible sources of infection. The liver-associated defect of the IVC after en bloc tumor resection is then reconstructed by the autologous graft of the IVC.

##### Surgical technique

The lengths of the caval segment that needs to be resected due to tumor infiltration are measured/estimated. After clamping of the lower and upper IVC, an appropriate segment from the infrarenal IVC is then resected and preserved in cold heparin/ sodium chloride solution on the back table.

This corresponding defect of the infrarenal IVC is reconstructed by the use of a ringed synthetic graft (e.g., GoreTex®). The synthetic graft is inserted by using a non-resorbable, monofile running suture (e.g., Prolene® 4/0). An omental flap is positioned around the synthetic interposition graft and the infrarenal IVC to separate this area from the peritoneal cavity (especially the liver).

Reconstruction of the corresponding defect of the IVC is also possible by the use of autologous peritoneum.

**Fig. 10:**
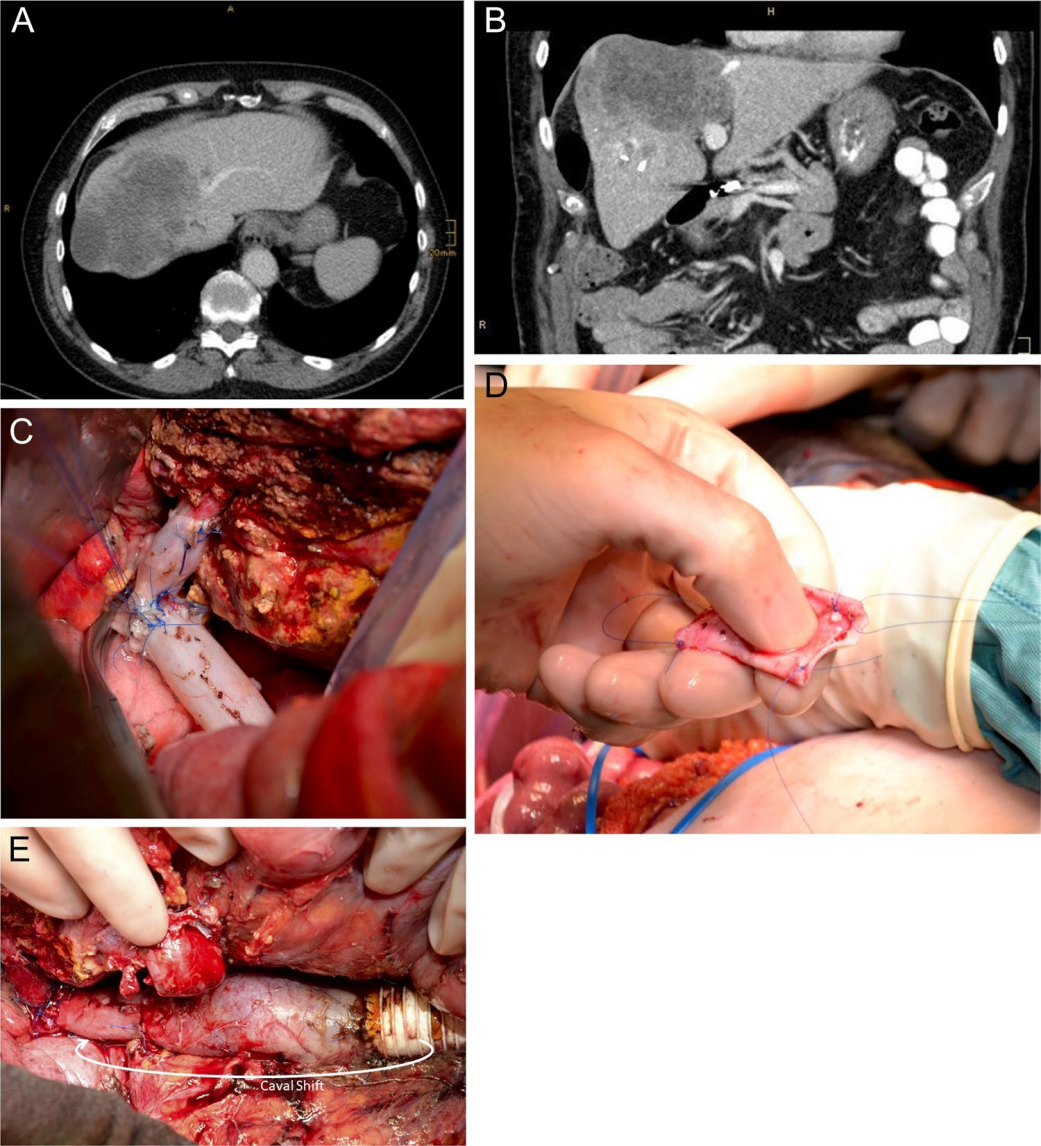
Patient with a cholangiocarcinoma infiltrating the vena cava and the right, middle, and left hepatic vein. Patient received extended right hemihepatectomy with reconstruction of the left hepatic vein by using a segment of the left internal jugular vein and reconstruction of the vena cava with a caval shift procedure as ante situm procedure. **a** and **b** Patient’s CT scan showing cholangicarcinoma infiltrating vena cava and all three hepatic veins. **c** Reconstruction of the vena cava after removing the tumor by extended right hemihepatectomy with insertion of an autologous caval segment. The left hepatic vein is reconstructed by interposition of left internal jugular vein. **d** Autologous segment of the infrarenal vena cava for reconstruction of the proximal IVC and venous confluence. **e** Caval shift procedure: adequate segment of infrarenal vena cava is resected and replaced by a ringed synthetic graft (e.g., GoreTex®). Picture shows the reconstruction of the infrarenal segment resection of the IVC on the right and the reconstructed IVC by the use of the autologous vena cava segment on the left

Afterwards, the defect of IVC after en bloc hepatic resection of the tumor and tumor-infiltrated IVC is reconstructed with the autologous caval segment obtained from patients’ own infrarenal IVC. The segment is inserted by using a non-resorbable, monofile running suture (e.g., Prolene 4/0). This procedure reduces infection of synthetic material in case of bile leakage or other postoperative infectious complications. The surgical technique of the caval shift procedure is shown in Fig. 10: a patient with cholangiocarcinoma of the liver infiltrating the right, middle, and left hepatic vein as well as the IVC. The patient was treated by extended right hemihepatectomy with the reconstruction of the IVC using the caval shift procedure as well as reconstruction of the left hepatic vein by using the left internal jugular vein (Fig. 10).

Caval shift procedure is recommended in case of higher risk for infectious complications related to liver resection.

### IVC resection with total vascular exclusion (TVE) of the liver

#### Resection of the IVC involving the IVC-HV confluence

Tumors that involve the IVC-HV confluence require the most demanding vascular resection in liver surgery. A total vascular exclusion (TVE) during resection is mostly necessary.

TVE was first described by Heaney in 1966 [114]. Besides technical aspects, the time of the vascular exclusion of the liver and the corresponding time of hepatic ischemia is the major challenge.

Tolerance of the liver with warm ischemia under TVE ranges between 30 and a maximum of 120 min [3]. The pre-operative assessment of the cardiac and pulmonary risk factors and renal function are essential issues in patient selection. Abnormality may increase the perioperative risk and morbidity significantly and should be considered a contraindication for extended resections [115].

During TVE, an inflow occlusion of the PV and hepatic artery as well as occlusion of the IVC above and below the hepatic veins is performed. This is considered to increase the degree of ischemic injury and must be kept as short as possible during resection [50]*.* Healthy liver parenchyma may tolerate over 60 min of inflow occlusion and warm ischemia [3]. For patients with damaged parenchyma or altered liver function, the tolerable ischemic time is mostly shorter [116].

The clamp placement should follow the sequence inflow before outflow (first hepatoduodenal ligament, second infrahepatic, and third suprahepatic IVC) [93]. Clamps are released after resection in reverse order, keeping in mind to partially release first the infrahepatic IVC to flush air out that might be trapped and avoid air embolism [93].

The pedicle and caval clamping during TVE leads to profound hemodynamic changes with a decrease in cardiac preload and output an increase in afterload. In a compensatory period of approximately 5 min after clamping, patients should be “preloaded” with intravenous fluids to maintain the blood pressure. If the middle arterial pressure (MAP) declines below 60 mmHg, TVE is not being tolerated, the caval clamps must be released, and a venovenous bypass should be considered. Furthermore, a renal dysfunction postoperative is often reported after TVE [117].

The consequence of these hemodynamic changes can be reduced by preconditioning measures as intermittent clamping and the use of anesthetic gases with vasodilatory properties like sevoflurane [117].

Moore et al. [118] described in 1960 in an animal model for liver transplantation the use of a venovenous bypass during the TVE period to maintain cardiac return and for portal venous decompression by cannulating the portal and femoral vein and the jugular and axillary veins. Venovenous bypass is used in a modified technique by some surgeons. Nevertheless, most of the patients tolerate the TVE also without venovenous bypass [51, 111, 119, 120].

In our own practice, the venovenous bypass wasn’t necessary in any case. However, transection of the liver parenchyma is mostly performed before the caval clamping for TVE, keeping this part of the operation as short as possible.

## In situ liver resection under in situ cold perfusion

Beside the patient’s cardiocirculatory stability and congestion of the small bowel under TVE, the main issue is the liver ischemia under complete vascular exclusion of the liver.

Learned from many experimental and clinical trials in liver transplantation, it is well known that warm ischemia is tolerated for a significantly shorter time than cold ischemia of the liver. Maximum tolerable warm ischemic time for the liver is about 60 min (range 30–120 min) [3, 119]. Fortner et al. [121] suggested that in situ hypothermic perfusion may reduce ischemic liver injury in cases when TVE is performed longer than 60 min. The liver is perfused by 4–8 L of the cooling solution at a temperature of 4 °C. The technique was first described by Fortner et al. [121] in 1974 in New York without using a venovenous bypass. He used Ringer’s lactate solution for in situ cold perfusion with a maximal cold ischemia time of 2h13min. The mortality rate of 29 hepatectomies under in situ cold perfusion was 10.3% [121].

For in situ cold perfusion of the liver, specific organo-protective solutions such as the HTK (Histidine-Tryptophan-Ketoglutarate)-Brettschneider or UW (University of Wisconsin) solution that contain specific cell-protective substances that are routinely used in liver transplantation are available.

Kim et al. [122] showed in a randomized-controlled trial reduced injury of the liver under hypothermic perfusion during TVE in humans.

This technique enables a comfortable resection in a bloodless field with very good visualization of the anatomic structures and with a reduced pressure of time.

In situ cold perfusion is recommended for complex liver resections with vascular reconstruction mostly of the hepatic veins/IVC requiring 1 h or more of TVE.

### Surgical technique

**Fig. 11:**
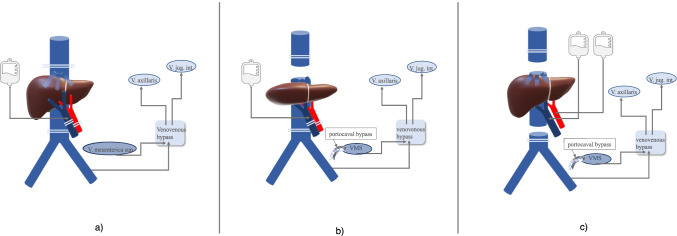
Principles of in situ and ex situ cold perfusion under TVE (venovenous and portalvenous bypass is optional in all procedures). **a** In situ cold perfusion: in- and outflow occlusion of the liver is performed. A cold perfusion solution is infused through the PV and drained via IVC into the abdomen. A venovenous bypass can be placed. **b** Ante situm resection under cold perfusion: Dividing the suprahepatic IVC allows better lifting of the liver and visualization of the venous confluence. **c** Ex situ resection under cold perfusion (modified from [93]): the liver is completely removed from the patient and cold perfusion is applied on the back table

After systemic application of 3000 IE heparin, total vascular exclusion of the liver is obtained by clamping of the portal vein and the hepatic artery as well as complete infrahepatic (above the renal veins) and suprahepatic (above the IVC-HV junction) clamping of the IVC. A catheter is then inserted into the portal vein and cold perfusion with preservation solution (HTK or UW solution) is started. The solution is drained into the abdomen via cavatomy of the infrahepatic IVC. In addition, the liver can be partially covered by sterile crushed ice to support the low temperature of the organ (Fig. 11a).

After completion of the reconstruction, the catheter is removed from the portal vein and the portal vein defect is closed by primary suture. Revascularization of the liver starts with the restoration of the inflow (portal vein and hepatic artery). The cavotomy is then closed by a running suture, and blood flow is restored after retro- and antegrad flushing and local application of heparin/saline solution.

Depending on the duration of ischemia, blood loss via cavotomy by open inflow and closed outflow with acceptance of a certain blood loss is often performed. Some surgeons accept a higher blood loss to reduce the risk of severe cardiocirculatory problems after wash in of electrolytes and other metabolites in the circulation after the clamping period. Some authors suggest flushing the liver with albumin before reperfusion to wash out the preservation solution [49].

Before reperfusion of the liver, good communication with the anesthesiologist is mandatory.

Even though liver surgery under in situ cold perfusion technique allows a bloodless situs and reduced time pressure, the access—especially to the hepatic veins—remains limited. In addition, the duration of in situ cold perfusion longer than 2 h is associated with systemic hypothermia of the patient [123]. Consequently, in situ cold perfusion techniques were further developed to ante situm and ex situ cold perfusion technique for better exposure and accessibility to the vessels.

In our center, cold perfusion of the liver is only applied in ante situm resections or ex situ resections of the liver.

## Ante situm liver resection under in situ cold perfusion

The ante situm liver resection technique describes a complete mobilization of the liver from the vena cava and positioning the liver in front of the abdomen rotating the liver completely up onto the abdominal wall, but still connected with the portal vein, hepatic artery, and bile duct.

Therefore, the complete hepatic venous confluence of the liver needs to be dissected from the IVC (including or excluding an IVC segment) and the liver needs to be completely detached from the vena cava (avoiding tumor infiltrated segments of the vena cava). This procedure is performed under TVE and in situ cold perfusion of the liver.

This method was first described by Hannoun et al. [124] in 1991 as “in-vivo-ex-situ” procedure. The rotation of the liver out of the abdomen allows better access to the hepato-caval confluence for the reconstruction of the hepatic veins. Most authors use this technique if combined IVC- and HV- resection and -reconstruction is required [125]. The use of a venovenous bypass during this procedure is described [51]. Most authors perform this technique without venovenous bypass as most of the patients tolerate the caval clamping without difficulty if they are sufficiently volume-loaded [4, 126–128]*.* The advantage of this approach is that hilar structures are not divided albeit to compare the access unfavorably to ex vivo resection.

### Surgical technique

After systemic application of 3000 IE heparin, total vascular exclusion of the liver is performed. In cases, with reconstructions of the IVC-HV confluence, the IVC needs to be completely mobilized and complete infrahepatic (above the renal veins) and suprahepatic (above the IVC-HV junction) clamping of the IVC, clamping of the portal vein, and the hepatic artery is necessary. The IVC is then dissected above and below the IVC-HV confluence and the liver is then rotated ante situm.

In the case of isolated HV reconstruction without IVC reconstruction, tangential resection of the HV confluence is possible with tangential clamping of the IVC. Hemming et al. [129] favorized mobilization of the liver in piggy-back technique as performed during liver transplantation. However, mobilization of the IVC is recommended to obtain bleeding control.

The liver is ante situm packed in sterile crushed ice and a catheter is then inserted into the portal vein and cold perfusion with preservation solution (HTK or UW solution) is started. The solution is drained via hepatic veins/resected IVC segment (Fig. 11b).

Liver parenchyma can be transected before performing the ante situm step and cold perfusion of the liver. However, some surgeons prefer to perform parenchyma dissection ante situm as this is felt to be easier and may result in a lower blood loss. Noteworthy, that one should be aware of the risk of bleeding from livers transection plane after reperfusion.

After ante situm reconstructions of the hepatic vein(s), HV-IVC confluence is reinserted into the IVC in situ. Before completion of the IVC anastomosis, the catheter is removed from the portal vein and the portal vein defect is closed by primary suture. Revascularization of the liver starts with the restoration of the inflow (portal vein and hepatic artery). After flushing and local application of heparin/saline solution, blood flow is restored and IVC anastomosis completed.

Depending on the duration of ischemia, higher blood loss by longer flushing by open inflow and closed outflow is performed by some surgeons as well as flushing with albumin to minimize reperfusion problems. Again, good communication with anesthesiologists is essential before reperfusion of the liver.

In the case of venovenous bypass, after TVE a catheter is inserted into the liver distant part of the portal vein and connected to the left jugular vein. In addition, a second catheter connects the femoral vein with the jugular vein. Venovenous bypass can help to overcome cardiocirculatory problems as well as congestion of the intestine during the TVE period [49, 129].

In our center, we perform ante situm resections usually by the use of HTK solution for cold perfusion, and the liver is packed with sterile crushed ice to support cold perfusion. A venovenous bypass has never been necessary in our center.

## Ex situ liver resection under ex situ cold perfusion

For ex situ liver resections, the liver is completely taken out of the patient. Resection is performed while perfusion with cold preservation solution on ice on the back table is applied. Besides the HV confluence, the hilar structures as the portal vein, hepatic artery, and bile duct need to be cut through as well.

This technically demanding procedure was first described by Pichlmayr et al. [130, 131] in 1988*.* The technique was developed using the experience gained in liver transplantation, for performing surgery of advanced tumors with involvement of the hepatocaval confluence and hilar structures. Since then, few authors have reported results from small series of several patients who underwent this procedure. The advantage of the ex situ resection is the excellent exposure and accessibility for performing complex resections and intrahepatic vascular reconstruction which enables tumor negative margins. During the procedure on the back table a venovenous bypass can be implemented or an IVC graft interposition (Gore-Tex) with a temporary portocaval shunt attached to avoid splanchnic congestion.

The steps of the resection described by Pichlmayr et al. [131] obtain the assessment for resectability of the tumor, followed by dissection of the hilum, infra- and suprahepatical IVC, and mobilization of the liver. Hilar structures and IVC are transected and the liver is removed and placed in an ice bath. Cold perfusion with preservation solution is started. After completion of resection and reconstruction, the liver remnant is reimplanted [131]. Long-term results of the series of ex situ liver resections by Oldhafer and colleagues [106] showed mean cold ischemic time of the liver of 5.6 h + /1.1 h (range 4–9 h).

### Surgical technique

After complete mobilization of the liver, total hepatectomy is performed after systemic application of 3000 IE heparin. As described above, the hilar structures are clamped first and transection of the hepatic artery, the portal vein, and the bile duct is performed.

Depending on the extent of tumor infiltration, hepatic veins are either resected with or without IVC segment. In case of segmental resection of the IVC, reconstruction of the IVC by using autologous or synthetic graft interposition can be performed immediately after hepatectomy to restore blood flow of the IVC during the ex situ preparation of the liver.

The liver is than placed on a back table in a bath of preservation solution surrounded by crushed ice.

According to back table procedures during liver transplantation, the portal vein as well as hepatic artery is perfused with ice-cold preservation solution (HTK or UW solution). The solution is drained via hepatic veins/resected IVC segment into the bath (Fig. 11c).

Transection of liver parenchyma as well as resection and reconstruction of vascular structures is performed ex situ under continuous cold perfusion. After completion of liver resection and reconstruction, the liver is reimplanted. First, Reinsertion in the IVC, followed by anastomosis of the hepatic artery and the portal vein is performed. After reperfusion of the liver, re-implantation of the liver is completed by bile duct anastomosis.

Depending on the duration of ischemia, higher blood loss by longer flushing by open inflow and closed outflow is performed by some surgeons to minimize reperfusion problems. Again, good communication with anesthesiologists is essential before reperfusion of the liver.

## Oncological aspects and postoperative results after IVC-HV reconstruction

Indications for hepatectomies with IVC and /or HV reconstruction include all malignancies such as CRLM or other metastases, cholangiocarcinoma, or hepatocellular carcinoma.

Combined hepatectomies with IVC reconstruction are routinely performed in highly specialized centers with good postoperative results. Combined liver resections with HV or IVC-HV reconstruction are technically more demanding. In situ or ex situ cold perfusion techniques allow technically complex liver resections with vascular reconstructions.

Due to heterogeneity of patients’ cohorts with different indications for surgery as well as a wide variety of surgical procedures and vascular reconstructions, short- and long-term results show a large range.

According to the literature, postoperative morbidity varies from 25 to 64% and postoperative mortality ranges from 0 to 12%. Regarding long-term results of vascular reconstruction, patency of IVC grafts is reported between 89 and 100% for synthetic graft reconstruction of the IVC [103–112]. Pulitano et al. [49] reported a graft patency for autologous IVC reconstruction of 100%. HV reconstruction and combined HV-IVC reconstruction patency of vascular reconstruction are reported up to 10% (Table 3)*.*

**Table 3:** Studies reporting about liver resections with outflow reconstruction: inferior vena cava, hepatic vein, or combined vena cava-hepatic vein reconstruction. *IVC inferior vena cava; TVE total vascular exclusion; CRLM colorectal liver metastases; HCC hepatocellular carcinoma; CCC cholangiocarcinoma; iCC intrahepatic cholangiocarcinoma; RCC renal cell carcinoma; GIST gastroinestinal stroma tumor; HV hepatic vein; RHV right hepatic vein; PV portal vein*

Author	Year	Number of cases/indication	Mode of IVC resection	Technique of vascular reconstruction	Associated liver resections	In- hospital morbidity	In-hospital mortality	Survival rates
**IVC reconstruction**								
Miayazaki et al. [132]	1999	*n* = 16 CRLM: *n* = 14 Met. gastric cancer: *n* = 1 Met. uterine cancer: *n* = 1	*n* = 16 *n* = 8 TVE *n* = 3 in situ cold perfusion *n* = 5 IVC side clamping *All TVE* > *30 min with venovenous bypass*	*n* = 13 primarily closure *n* = 2 patches *n* = 1 Gore tube	*n* = 16 liver resections (12 major and 4 minor hepatectomies)	25%	6%	CRLM: 1-year 82% 5-year 27%
Sarmiento et al. [110]	2003	*n* = 18 CCC: *n* = 9 Metastases: *n* = 5 HCC: *n* = 2 Other: *n* = 2				5.5% (1 intraop. death)		5-year: 21%
Azoulay et al [133]	2006	*n* = 22 Liver metastases: *n* = 9 CCC: *n* = 8 HCC: *n* = 2 Others: *n* = 3	*n* = 22 IVC *n* = 1 TVE with preserved caval flow *n* = 12 TVE *n* = 9 in situ cold perfusion *No *ex situ* resections* *n* = 6 HV reimplantation	*n* = 10 synthetic grafts *n* = 8 primary suture *n* = 4 caval plasty		64%	4.5%	1-year 81.8% 5-year 38.3%
Malde et al. [102]	2011	*n* = 35 CRLM: *n* = 21 HCC: *n* = 6 CCC: *n* = 3 Other: *n* = 5	*n* = 35 IVC Without TVE: *n* = 1 TVE n = 34 Cold perfusion: *n* = 22 ➢ 13 in situ ➢3 ante situm ➢6 ex situ	*n* = 23 IVC patches *n* = 12 synthetic IVC grafts	*n* = 30 major hepatectomies	40%	11%	1-year 37.7% CRLM: 75.9% HCC: 83.3% CCC: 33.3%
Pulitano et al. [49]	2013	*n* = 32 CRLM: *n* = 5 Leiomyosarcoma: *n* = 8 RCC: *n* = 11 Adrenal cancer: *n* = 2 HCC: *n* = 1 iCC: *n* = 2 GIST: *n* = 1	*n* = 32 IVC TVE *n* = 10 with HV or RV reimplantation Patch if < 30% circumference > 40% segment	*n* = 22 segmental IVC graft *n* = 10 IVC patches *(n* = *10 bovine* *n* = *22 peritoneum)*	*n* = 14 liver resections *(7 major and 7 minor hepatectomies)* *n* = 10 nephrectomy *n* = 2 pancreatico-duodenectomy	28%	9%	1-year 78% 5-year 48%
Hemming et al. [51]	2013	*n* = 60 CCC: *n* = 26 HCC: *n* = 16 CRLM: *n* = 13 GIST: *n* = 2 Hepatoblastoma: *n* = 2 Squamus cell carcinoma: *n* = 1	*n* = 60 IVC *n* = 1 tangential cava clamping *n* = 8 in situ cold perfusion *n* = 6 with HV/PV reco *n* = 3 with open pericardium *All *ex vivo* resections with venovenous bypass*	IVC primarily 8 Tube graft 38 Patches 14	*n* = 60 major hepatectomies	43%	8%	1-year 89% 5-year 35%
**Hepatic vein reconstruction**								
Hemming et al [134]	2002	*n* = 16 HCC: *n* = 9 CRLM: *n* = 5 CCC: *n* = 1 Hepatoblastoma: *n* = 1	*N* = 16 HV (10 entire venous outflow; 6 reco of RVH additionally) *n* = 5 combined with IVC *n* = 1 in situ cold perfusion *n* = 2 ex situ with venovenous bypass	6 RHV (4 Gore) 10 major outflow (8 reimplanted, 2 using portal vein grafts)			12%	1-year 88% 3-year 50%
Saiura et al. [50]	2011	*n* = 16 (CRLM)	16 hepatectomies with HV reconstruction	18 HV recos Saphenous 10 Direct anastomosis 1 External iliac vein 2 Portal vein 2 Umbilical vein patch graft 3 Ovarian vein patch graft 1	*n* = 5 major liver resections *n* = 11 minor liver resections	50%	0	1-year 93% 5-year 76%

One of the largest series, published by Hemming et al. [51], included 60 patients undergoing hepatectomy with IVC resection for different malignancies, and reported perioperative mortality of 8% and postoperative morbidity of 43% within 90 days of surgery. Overall, 1-and 5-year survival rates were 89% and 35%, respectively [51]. Azoulay et al. [133] reported a cohort of 22 patients who received combined liver resection with retrohepatic IVC reconstruction for liver metastases, CCC or HCC. IVC reconstruction was performed under TVE in 12 cases, under vascular exclusion of the liver with preserved caval flow in 1 case and with in situ cold perfusion in 9 cases. Venovenous bypass was necessary in 12 cases. No ex situ resections were performed. Perioperative mortality was 4.5% and morbidity 64%. One-year and 5-year survival rates of 81.8% and 38.3% are encouraging results [133].

In another analysis, the same authors compared in situ cold perfusion techniques with standard vascular exclusion technique in liver surgery in 69 patients: 33 patients under TVE < 60 min, 16 patients under TVE > 60 min, and 20 patients receiving liver resection under in situ hypothermic perfusion of the liver were compared. Postoperative mortality varied between the groups with 1/33 in TVE < 60 min, 2/16 in TVE > 60 min, and zero mortality in patients resected under cold perfusion. In addition, patients with in situ cold perfusion showed significantly better tolerance to ischemia compared to the TVE groups. Significantly better postoperative liver and kidney function was observed compared to the patients with TVE > 60 min [3].

Table 3 summarizes important studies reporting about IVC and/or HV reconstruction of the last decades.

Compared with the in situ technique, ex situ resections are more challenging due to the division of hepatic pedicle and demand an expertise with liver transplantation techniques. Only a few studies published results from small patient cohorts or case reports.

Govil et al. [135] compared in a review about TVE the postoperative mortality in liver resections under in situ cold perfusion, ante situm resections, and ex situ resections. He reviewed 18 studies and included own experience and obtained postoperative mortality of 4.65% for in situ cold perfusion, 2.9% for ante situm resections, and 31.5% for ex situ resections [135].

Long-term results of those advanced surgical procedures depend not only on technical success, but also on tumor biology. However, long-term survival of 11–27% is reported, which appears acceptable for a small patient cohort considering the fact that without surgery the life expectancy for those patients is less than 1 year [86]**.**

## Anticoagulation strategies

Despite the increasing number of vascular reconstructions in liver surgery, there are no specific guidelines or studies for anticoagulant strategies for vascular surgery in liver resection. However, there are some recommendations for anticoagulant prophylaxis after arterial reconstruction in general vascular surgery [136], experiences and recommendations for anticoagulation after orthotopic liver transplantation [137, 138], and case reports about vascular reconstruction of the portal vein or reconstruction of the vena cava [109, 113, 139]. In addition, there are general guidelines for perioperative venous thromboembolism prophylaxis in general and visceral surgery [140]. According to the available literature and our own experience we defined some guidelines within our institution:

### Intraoperative anticoagulation:

Most authors apply heparin i.v. at doses between 3000 and 5000 IE before vascular clamping for vascular reconstruction [113, 141]. According to the guidelines within our institution, no anticoagulation is administered before cava clamping or pedicle clamping for in- and outflow control during liver resection. Noteworthy, clamping time of maximum 10 min (Pringle) and 15 min (VCI) per cycle is performed. Different from other centers, no systemic anticoagulation is administered before clamping for resection and reconstruction of the venous system (including partial or complete clamping of the IVC, the hepatic veins, or the portal vein). We didn’t experience any intraoperative or early postoperative thromboembolic complication in those patients.

Before clamping the arterial system (hepatic artery), 3000 IE of unfractioned heparin i.v. is used prior to clamping and reconstruction of the artery.

However, different from other authors, no intraoperative control of pTT or application of protamine for reversal of anticoagulation is routinely used after completion of the anastomosis.

According to general principles of vascular surgery, local application of a solution of heparin/saline (5000 IE heparin/500 nl 0.9% sodium chloride) is performed as well as flushing before the blood flow is restored for arterial and venous anastomoses.

### Postoperative anticoagulation:

For postoperative anticoagulation, therapeutic regimens vary widely between different authors and within different studies. The use of postoperative aspirin (ASS), low-molecular-weight heparin (LMWH), or unfractionated heparin (UFH) is described as well as no postoperative anticoagulation [113, 137, 138, 141].

In our institution, all patients after liver resections without vascular reconstruction as well as all liver resections with tangential or segmental resection of the vena cava do not obtain specific anticoagulant treatment and are treated following the German interdisciplinary, evidence- and consensus-based (S3) clinical practice guideline on venous thromboembolism prophylaxis based on patient-related risk factors, and type of surgery [140]. The patients receive pharmacological prophylaxis with heparin (low-molecular-weight LMWH or unfractionated UFH) for 4 weeks postoperative if no complication occurs.

After any reconstruction of the hepatic artery, postoperative treatment with ASS is recommended and maintained for 1–3 months depending on the size of anastomosis and quality of the reconstructed artery. In addition, intravenous heparin in the early postoperative days (POD 1–3) is applied aiming to reach a partial thromboplastin time (pTT) of 40–50 s. Treatment with intravenous heparin can be quickly corrected according to clinical course and bleeding events. For the further postoperative course, additional regular venous thromboembolism prophylaxis is performed.

After reconstruction of the portal vein by primary end-to-end anastomosis or by the use of an autologous interposition graft, either regular venous thromboembolism prophylaxis with LMWH or intravenous heparin with pTT aiming to reach 30–40 s. on POD 1–3 is applied. Further postoperative anticoagulation depends on the quality and diameter of anastomosis. In regular anastomosis, LWMH is applied in prophylactic doses. In the case of small or waisted anastomoses, LWMH is applied in half-therapeutic doses.

After any reconstruction (portal vein, hepatic vein, IVC) with a synthetic graft interposition, therapeutic anticoagulation with intravenous heparin with a pTT aiming to reach 50–60 s is used for POD 1–3 and continued with fully therapeutic anticoagulation with oral anticoagulants (coumarone) for 3 months, considering higher risk for thromboembolism. Although the reported incidence of postoperative thrombosis in cases of graft use is not significantly higher, some authors prefer the use of systemic anticoagulation after surgery with new oral anticoagulants (NOAC) [109, 139].

In our department, early imaging of complex vascular reconstruction by contrast-medium-enhanced CT scan is performed to display the quality of anastomosis and to adapt anticoagulation if necessary.

Appropriate use of postoperative anticoagulation therapy is still under debate and no defined standards are established in current literature and praxis. The optimal antithrombotic management remains a clinical decision based on the type of surgery, patient condition, and use of grafts, but the value of LMWH is widely accepted [136].

## Conclusion

Malignancies of the liver infiltrating even central vascular structures are nowadays not a contraindication for surgery in specialized centers. Due to the development of multimodal concepts and technical improvements in liver surgery, reconstruction of the portal vein and the hepatic artery as well as hepatic veins (with or without the IVC) combined with major hepatic resections is successfully performed. Techniques learned from liver transplantation allow cutting-edge ante situm or ex situ liver resection under hypothermic organ perfusion.

Noteworthy, highly complex cases/procedures are still associated with high morbidity and relevant mortality. Good patient selection is important to improve not only patient’s survival, but also the quality of life. Considering the fact that surgery is the only curative option for advanced hepatic tumors, extended hepatectomy with vascular resection is justified as a curative approach. This message should not only be known in specialized centers, but should also infiltrate oncological boards to show up the possibility of surgery for those patients.

Even though vascular surgery in liver resection is routinely performed in specialized centers worldwide, no clinical trials or guidelines are available regrading peri- and postoperative anticoagulant strategies. Clinical trials supporting to define guidelines for those procedures are needed.
